# Coastal marine habitats deterioration according to users’ perception: the case of Cap de Creus Marine Protected Area (NE Spain)

**DOI:** 10.1007/s10113-024-02322-4

**Published:** 2024-10-10

**Authors:** Miguel Mallo, Patrizia Ziveri, Sergio Rossi, Victoria Reyes-García

**Affiliations:** 1 Institut de Ciència i Tecnologia Ambientals (ICTA), Universitat Autònoma de Barcelona (UAB), Bellaterra, Barcelona, Spain; 2 Institució Catalana de Recerca i Estudis Avançats (ICREA), Barcelona, Spain; 3 Departamento de Biología Animal, Biología Vetegetal i Ecología, Universitat Autònoma de Barcelona (UAB), Bellaterra, Barcelona, Spain; 4 Dipartimento Di Scienze e Tecnologie Biologiche e Ambientali (DiSTeBA), Università del Salento, Lecce, Italy; 5 Labomar, Universidade Federal Do Ceará, Fortaleza, Brazil; 6 Department Antropologia Social i Cultural, Universitat Autònoma de Barcelona (UAB), Bellaterra, Barcelona, Spain

**Keywords:** Environmental change, Local knowledge, Marine ecosystem, Mediterranean Sea, Perceptions of change, Tourism

## Abstract

Assessing how different users of a Marine Protected Area perceive environmental changes can contribute to design management strategies. We assess how locals and tourists perceive environmental changes in the Cap de Creus protected area (NW Mediterranean, Spain). To identify locally perceived changes, we first conducted semi-structured interviews with locals (*n* = 38). Reported environmental changes were then used to construct a survey applied to locals and tourists (*n* = 427). In semi-structured interviews, environmental changes were the least reported changes compared to economic and social changes; reports of negative environmental changes dominated over reports of positive environmental changes. Overall, all survey participants reported a decline of the health status of the local environment, with locals displaying higher levels of agreement with statements referring to coastal environmental deterioration than tourists. The predominance of responses reporting economic versus environmental changes can be explained by the recent radical transformation of the area towards tourism. Reports of coastal marine area deterioration are in line with available instrumental data. Higher levels of agreement with statements referring to environmental deterioration among locals than among tourists highlight the importance of people’s connection with nature to assess change. Considering that Cap de Creus has been protected for more than two decades, our findings raise concerns regarding its ongoing deterioration and underscore the importance of monitoring the effectiveness of marine protection to modulate management strategies.

**Supplementary Information:**

The online version contains supplementary material available at 10.1007/s10113-024-02322-4.

## Introduction

Human perception of the surrounding environment provides a limited representation of reality. Through senses, humans are only able to process a small fraction of all the stimuli around, which is then internally interpreted, resulting in a subjective and limited representation of reality (Gärling & Golledge 1989; Silva et al. 2016). Moreover, environmental perception, understood as the “physical aspects of the reception of visual stimuli, the intuitive recognition of an aesthetic quality and the ability of the mind to connect sensory information to other knowledge and so to develop opinions about what has been perceived” (Bell 2001, p. 202), is necessarily influenced by exposure to the environment. Therefore, it is not surprising that, given that marine ecosystems are less viewed, experienced, studied, understood, and appreciated than terrestrial ones (Gray 1997; Steel et al. 2005), we know little about people’s perceptions of marine environments and the changes affecting them. For example, Tonin and Lucaroni (2017) reported that 58% of a sample representative of lay people of major Italian cities had never heard about biodiversity on the coralligenous habitats and that very few had directly experienced being close to them.

While previous research on perception of coastal marine habitats (CMH) is scant, it has covered several aspects. Some previous studies on the perception of CMH status have focused on beach users’ perception of litter and dirtiness, concluding that local users perceive tourism as an important factor contributing to dirtiness (Garcés-Ordóñez et al. 2020; Hayati et al. 2020; Marin et al. 2009; Rayon-Viña et al. 2018). Other works have examined the perception of the effectiveness of protected areas conservation measures and/or the potential or perceived impact of conservation measures (Bennett & Dearden 2014; Engel et al. 2014; Leleu et al. 2012). Additional work has examined users’ perceptions of CMH ecosystem services (Blasiak et al. 2015; Blayac et al. 2014; Cárcamo et al. 2014), health status, ongoing impacts, and future threats (Jefferson et al. 2014; Mallo et al. 2022).

Additionally, and in the quest to inform historical ecological reconstructions, interest has also grown in understanding local perceptions of environmental changes, which could provide information on habitats functional transformation and the associated loss of ecosystem services (Lotze & Worm 2009; Shackeroff et al. 2011; Thurstan et al. 2015). Previous work analyzing perceptions of change in marine habitats has focused on changes in specific elements, such as commercial or culturally important marine species (Azzurro et al. 2011; Coll et al. 2014; Maynou et al. 2011; Pita et al. 2013), increase of plastic pollution (Arulnayagam 2020), physical changes on coastal areas (Boyer-Villemaire et al. 2014), or inhabitants’ changes of “sense of place” (Rogan et al. 2005).

A commonality in this body of research is that researchers agree that environmental perception is influenced by sociocultural attributes of informants (e.g., age, gender, education, or livelihood activity) (Howe & Leiserowitz 2013; Reyes-García et al. 2024; Silva et al. 2016), which can result in ambivalent and multi-facetted environmental appreciations. This is, for example, the case of peatlands in Scotland, which are simultaneously perceived as “good,” “bad,” and “ugly,” different perceptions depending not only on biophysical characteristics, but also on trade-offs between different uses and different personal relations with nature (Byg et al. 2017). Indeed, several works analyzing CMH perceptions have assessed variations in reported perception across people with different characteristics, comparing—for example—perceptions of people in different age groups or perceptions of locals vs. tourists (Abecasis et al. 2013; Tran et al. 2002). For instance, a study on a coastal region of Australia found that young tourists perceived recreational activities as more harmful than older tourists (Priskin 2003) and a study in Asturias (Spain) showed that young beach users perceived more litter on the beach than users of any other age group (Rayon-Viña et al. 2018). Nevertheless, researchers have not always found differences across groups. For example, in a study in Cap the Creus Marine Protected Area (MPA), researchers found that local inhabitants and tourists equally valued MPA regulating services and that both groups considered that marine pollution, climate change, and people’s behavior towards nature are important ongoing and future threats to the area (Mallo et al. 2022).

Previous work has noted that understanding the individual characteristics that pattern environmental perception can help understand behavioral differences in people’s relation to the environment (Spence et al. 2011; Weber & Johnson 2009) and contribute to improve environmental management. This is particularly the case in areas where two worldviews are confronted, as in highly touristic settings (Llausàs et al. 2019). For example, a study in Permuteran reefs (Bali, Indonesia) found that local people and those who interacted more with the sea contributed more to a coral reef restoration project, arguably because high level of interactions affects the way people think about whether reef ecosystems should be restored or not. The study also found that the community’s positive perception and participation in the restoration project was largely motivated by a group of local Balinese who were able to translate the need to protect reef ecosystems into the local language by associating it with spiritual beliefs and by giving the idea that restoring and conserving nature is the best investment for the future. According to the authors, building positive perceptions led to a high level of community participation to support the project (Trialfhianty & Suadi 2017).

Here, we contribute to this body of knowledge. Specifically, we extend our previous line of inquiry comparing local inhabitants’ and tourists’ valuation of ecosystem services and threats (Mallo et al. 2022) and assess how these two groups perceive changes in the Cap de Creus MPA in NE Spain (NW Mediterranean Sea). Given that environmental change is only one of the multiple changes being experienced in the area, our work starts by assessing which changes are observed in the area, including both positive and negative changes, as well as environmental, social, and economic change. We then evaluate differences in perceptions of change comparing responses from locals (i.e., residents of the area) and tourists (i.e., Cap de Creus visitors living more than 120 km away from the area) and examining whether there are other individual characteristics of locals and tourists that pattern environmental perception. We focus on Cap de Creus because it holds some of the richest biodiversity in the Mediterranean region, while also facing some of the highest levels of anthropogenic pressures (Bianchi et al. 2012; Claudet et al. 2020; Coll et al. 2012; Lejeusne et al. 2010). Cap de Creus MPA also provides an ongoing example of marine biodiversity conservation combined with active use for fishing and recreational activities (Corrales et al. 2020).

## Methods

### Study area

Cap the Creus MPA is in one of the most productive coastal regions of the Mediterranean Sea thanks to river runoffs and strong wind-induced vertical seawater mixing and surface sea fertilization (Rohling et al. 2015). Cap de Creus’ CMH lay mainly on sandy or rocky substrates, where seagrass meadows and coralligenous develop (Sardà et al. 2012). Both habitats, if healthy (Di Camillo et al. 2023; Montefalcone 2009), are hotspots of marine biodiversity (Duffy 2006; Valisano et al. 2019).

**Fig. 1:**
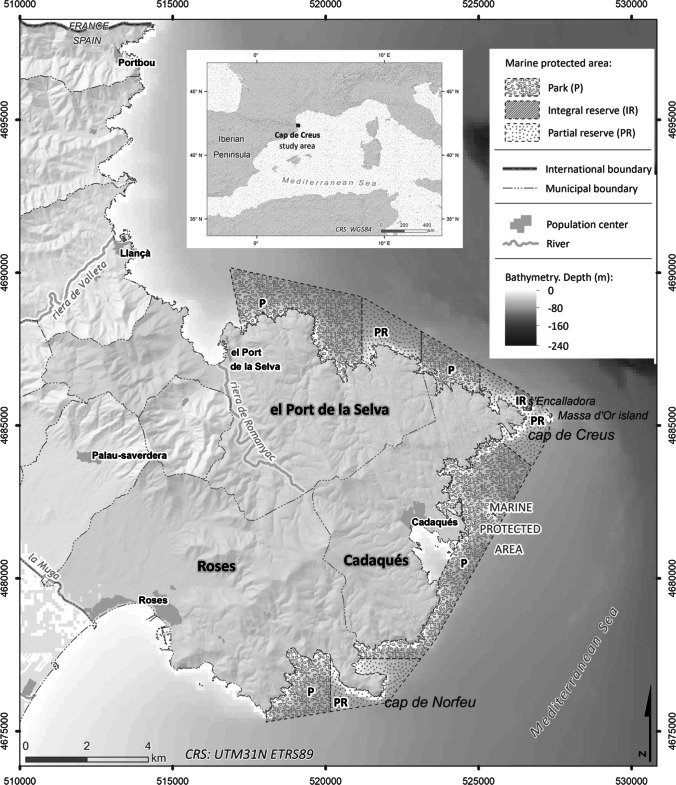
Map of the study area

The study area includes three municipalities of the Alt Empordà (Girona, NE Spain): Roses, Cadaqués, and El Port de la Selva, which are part of Cap de Creus MPA created in 1998 (Fig. 1). Traditionally, inhabitants of the three municipalities relied on small and artisanal fishery for subsistence and on the fishing industry (Gómez et al. 2006). The Costa Brava (including Cap de Creus) became an important touristic destination in the 1960s. Before that period, the main economic activities of the area were artisanal and small-scale fishing and agriculture (Gómez et al. 2006). Roses is the largest and most densely populated municipality of the study site, with 19,984 inhabitants in 2023, a population largely devoted to the tertiary sector (Idescat 2023). The site of Roses has had a strategic geopolitical position throughout history. Since the 1960s, it became a largely tourism-oriented municipality, with numerous touristic infrastructures and services. Nowadays, “sun-and-beach” and family tourism are Roses’ main income source. Cadaqués (pop. 2916) was traditionally a fishing town, although nowadays main occupations also come from the tertiary sector (Idescat 2023). Due to its gastronomy, architecture, and to the presence of several recognized artists during the twentieth century, Cadaqués largely attracts cultural tourism. Among the three municipalities studied, El Port de la Selva is the least densely populated, with a population of 1044 in 2023 (Idescat 2023). El Port de la Selva also had an important fishing tradition until the 1960s, when the economic activity of the area became dominated by tourism.

### Data collection

We collected data through semi-structured interviews and a survey. During December 2017 and July 2018, we collected qualitative data using semi-structured interviews. We then used the results of these interviews to design a survey that was used to collect data with a different sample during July and August 2019. The Ethics Committee of the Autonomous University of Barcelona approved our sampling procedure and data collection protocol (CEEAH 4792).

We conducted semi-structured interviews to obtain a list of the main changes in the study area perceived by people economically dependent on the local CMHs. To select participants, we gathered from the tourism offices of each municipality a list of owners and managers of tourist lodging, fresh seafood restaurants, and scuba diving centers. We contacted all the businesses listed (*n* = 93), although less than half replied to us (*n* = 38, answer rate = 43%; Online Resource 1), potentially resulting in self-selection bias in responses to semi-structured interviews.

We requested informants to report both positive and negative changes observed in the study area. This includes social changes (or transformation of social structures, relationships, behaviors, and community dynamics over time, such as population decline or aging), economic changes (or alterations in the way local economies function, including shifts in employment patterns, industries, income levels, infrastructure, and access to markets, such as job seasonality or shift to no-fishing jobs), and environmental changes (or transformation of the natural landscape and ecosystem due to human activities or natural processes, including changes in climate, biodiversity, and the availability of natural resources). Specifically, semi-structured interviews were organized around two questions. First, we asked: «*What do you think has changed negatively / to the worst since you started doing your activity in this area until today? Consider things that have affected both you and other town citizens*». Then, we asked the opposite, i.e., using the same question but changing «*negatively/to the worst*» by «*positively/to better*». We hand-noted answers.

Answers to semi-structured interviews were used to construct a survey to assess perceptions of local environmental changes. The sample for the survey included locals, or people who reside in the study area or nearby towns, and tourists, or Cap de Creus visitor who live 120 km or further from the study area (Table 1). To select participants, we used a convenience sampling strategy consisting of approaching adults in the streets of Roses, Cadaqués, and El Port de la Selva and requesting their participation in our survey. For people who initially agree, we first checked whether they had known the area for at least 10 years, a prerequisite for participation. We approached 1227 people, 65% of them declined to be interviewed or did not meet the prerequisite. The final sample for the survey is of 427 people (Online Resource 1).

**Table 1:** Sociodemographic variables used in the analysis

Survey question	Variable	Codes
Town/city of residence	Local^a^	Local: Resident in Roses, Cadaqués, El Port de la Selva, Cala Jòncols, Castelló d’Empúries, Empuriabrava, Llançà, Selva de Mar, and Vilajuïga
		Tourists: Cap de Creus visitor living more than 120 km away from the area
Town/city of residence	Residence-to-sea distance	Coastal: Residents of a town/city that limits with the sea
		Near coast: Living at less than one hour drive to the closest coast by car (calculated with © Google Maps)
		Inland: Living an hour’s drive, or more, drive from the closest coast
To what extent do you depend on the marine environment in your daily life, either for your work and/or income or for your leisure activities?	Marine affinity/dependence	Dependent: “Strongly. I need it frequently” and “I need it once in a while, I cannot spend too much time without it.”
		Joy: “I enjoy it, but I do not depend on it.”
		Indifferent: “Indifferent. It has nothing to do with me.”
		Avoid: “I avoid it.”
What is the highest level of education that you have completed?	Education level	Low: No schooling completed, Primary school, and Middle school
		Medium: High school and Professional degree/technical school training
		High: University, Master, post-graduate and PhD
Which category best indicate your average yearly income?	Income level	Low: < 14,000 €/year
		Medium: 14,000–35,000 €/year
		High: > 35,000 €/year
What is your age group?	Age group	Young: 18–29 years
		Middle: 30–49 years (30–39 and 40–49 grouped)
		Older: > 50 years (50–59, 60–69, 70–79, 80–89, > 90 grouped)
What was the first decade when you visited the area?	Baseline decade	Before 1970: People born before 1970
		1970s: People born from 1970 to 1979
		1980s: People born from 1980 to 1989
		1990s: People born from 1990 to 1999
		2000s: People born from 2000 to 2009
		2010s: People born from 2010 to 2020

^a^Respondents living closer than 120 km but who are not locals are not included in the analysis

The survey collected respondent’s sociodemographic information (e.g., place of residency, dependence of CMH, education) and a level Likert scale with 10 statements about local environmental changes. Likert-scale statements were derived from information on semi-structured interviews. For each statement, respondents had to indicate their level of agreement with the statement, where 0 = N/A, 1 = Strongly disagree, 2 = Disagree, 3 = Neither agree nor disagree, 4 = Agree, 5 = Strongly agree (Online Resource 2). To answer questions about change, we asked locals to consider the status of the area during their childhood as baseline and visitors to consider their first visit to the area.

### Data analysis

We created two lists of changes observed in the area by grouping responses from semi-structured interviews referring to the same change. One list included changes perceived as positive and the other list included changes perceived as negative. Each item in the list was also classified as social, economic, or environmental change. We calculated the number of interviewees reporting each change in our lists.

Before analyzing survey data, we recoded responses to Likert-scale assessments. First, we transformed “N/A” (= 0) responses into “Neither agree nor disagree” (= 3), as many informants misunderstood these categories making their differentiation impossible. This reduced our scale from a 6 to a 5-point scale. Second, we reversed the code of some responses. To minimize response bias by automation, in some survey questions, a score of 5 indicated agreement with a deterioration of the area, but in other questions agreement with deterioration corresponded with a score of 1. We reverse the code from answers to these statements so a score of 5 indicates strong agreement with a deterioration of the CMHs compared to the past.

We then used survey results to analyze perceptions of change for the whole sample and comparing responses of locals vs. tourists. We used the numerical value of responses to the Likert scale to compare the mean value of the ten statements across samples. In the final part of the analysis, we created subsamples that included additional sociodemographic characteristics, beyond just distinguishing between locals and tourists. Such characteristics include (i) residence-to-sea distance, (ii) dependence of CMHs, (iii) age group, (iv) education level, (v) income level, and (vi) baseline decade (see Table 1 for variable definition). To test whether there are statistically significant differences between locals’ and tourists’ perceptions of CMH environmental change, while also considering these sociodemographic characteristics, we used a Kruskal–Wallis rank-sum test (*α* = 0.05) and a post hoc Dunn test with the Holm method for *p*-values adjustment. We used R-Studio v1.2.5033 for all analyses.

## Results

Our sample for semi-structured interviews was unbalanced towards men (64%). Respondents had an average of 51 (± 12) years of age and had been working in the area for an average of 31 (± 14) years. Respondents reported 63 different changes: 24 positive and 39 negative. Most reported changes referred to economic aspects (41%), followed by social (30%) and environmental aspects (29%). Economic changes were reported by 47% of people interviewed, whereas social and environmental changes were only reported by 29% and 24% of respondents, respectively (Online Resource 3). Overall, the frequency of changes reported as negative (75% of all changes; 7.2 per interview) was higher than the frequency of changes reported as positive (25%; 2.4 per interview). The distribution between positive and negative changes is similar for the pooled set of responses than when differentiating among economic, social, and environmental changes.

Overall, respondents reported 88 instances of environmental changes, which correspond to 18 different changes, of which 12 were considered a deterioration (e.g., less biodiversity, more boats and anchoring, more invasive species) and six were considered an improvement of the CMH (e.g., more biodiversity, better beach/coast) (Table 2). Reports of negative environmental changes were more frequent, representing 70 of the 88 reports (79.5%; 1.8 per interview). The two negative environmental changes most often reported referred to biodiversity deterioration (16 reports) and increase of disrespectful users (9 reports). There were 18 reports of positive environmental changes (0.5 per interview), although 24 respondents (63.2%) did not report any positive environmental change. The positive environmental change most often reported referred to biodiversity increase (8 reports) (Table 2).

**Table 2:** Frequency of reports of positive (improvement) and negative (deterioration) environmental change from semi-structured interviews (*n* = 38)

	Improvement	Deterioration
Marine life	8	16
Beach/coast condition	3	5
Impacts from construction	3	
Impacts from sewage plants	2	
Aesthetic value	1	3
Respect towards nature	1	9
Natural Park efficiency		6
Overfishing		6
Impacts from tourism		6
Impacts from boats and anchoring		5
Presence of invasive species		5
Species health		2
Marine pollution		1

**Fig. 2:**
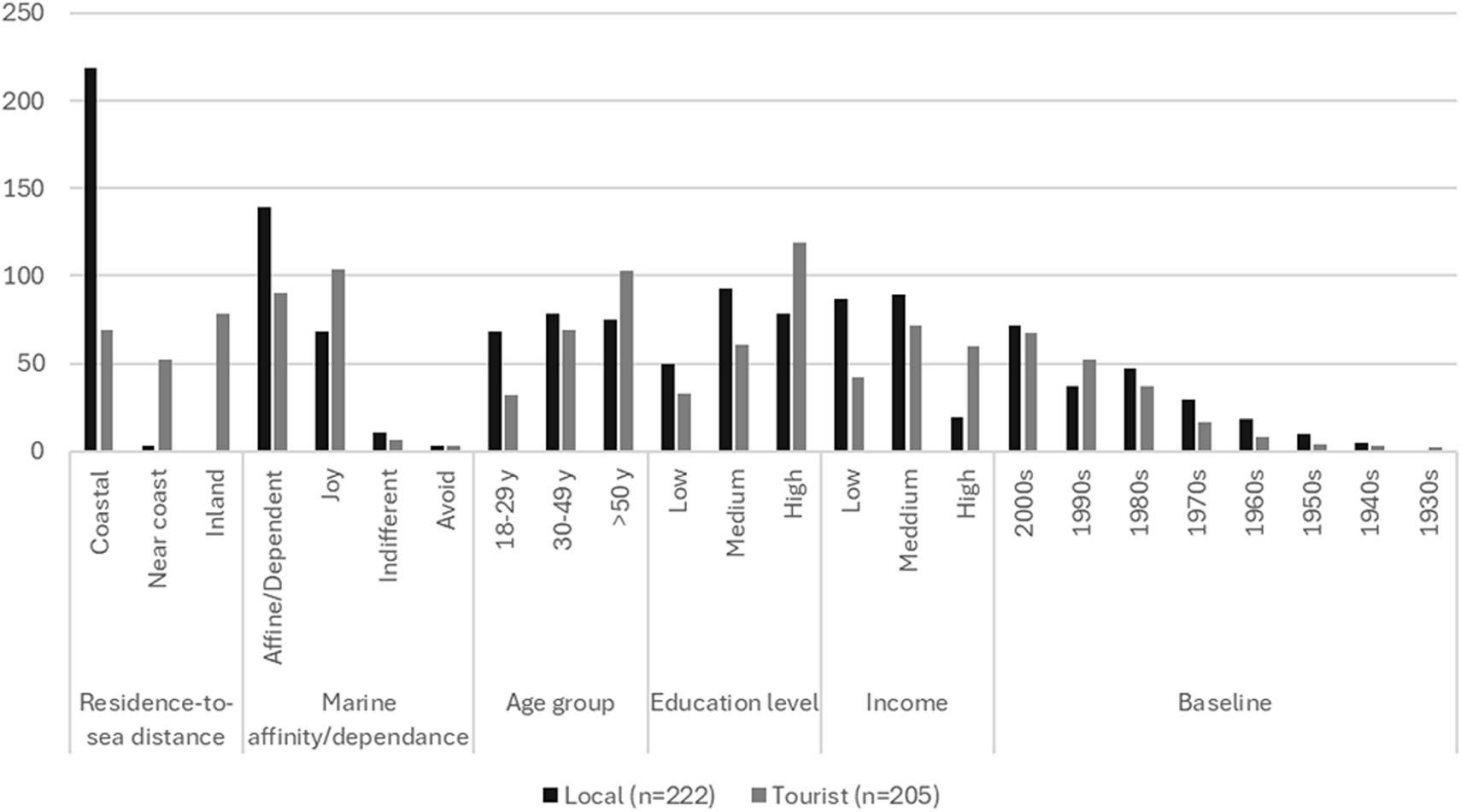
Number of local and tourist survey respondents, by sociodemographic characteristics (*n* = 427)

Given our sampling design, the survey sample (*n* = 427) was homogeneously distributed across the three municipalities (Cadaqués 33.7%, El Port de la Selva 33.7%, and Roses 32.6%) and between locals (52%) and tourists (48%; Online Resource 4). Most people in our sample lived in coastal areas, although the distribution was biased between locals, who all lived in coastal areas, and tourists, who resided in similar proportions in coastal, near coastal, and inland areas (Fig. 2). Most locals had a strong affinity with and/or dependence on CMHs, while most tourists stated that their relationship with CMHs corresponded to the joy and dependence categories. Respondents in our sample were spread across age groups, but tourists slightly dominated the category of 50 years of age and above. The level of education of people in our sample was generally high, although education levels were lower among locals than among tourists. Most people in the sample had an income between 14,000 and 35,000 €/year. Tourists dominated in the higher income categories (> 35,000 €/year), whereas locals dominated in the categories under 35,000 €/year. Finally, there was a predominance of people with a reference baseline on the 2000s and a low proportion of the sample with a reference baseline on the 1970s and before (Fig. 2).

Across the sample, the statements for which most respondents “strongly agree” were the ones referring to the fact that there is “less marine life” (56.4%), “more marine pollution” (45%), and “more boat transit and anchor” (41.9%) now than in the past (Online Resource 5). Around a third of the sample “agree” that now there is “more tourism impact” (37%), “nostalgia of a more beautiful sea” (33.7%), “overfishing” (35.8%) and “hotter temperatures” that in the past (35%). The most frequently selected option for the statement that there are “more invasive species” now than in the past was “neither agree nor disagree” (40.8%). Finally, most informants “disagree” that there is “less respect towards nature” (31.1%) and “less clean coasts” now than in the past (31.4%).

**Fig. 3:**
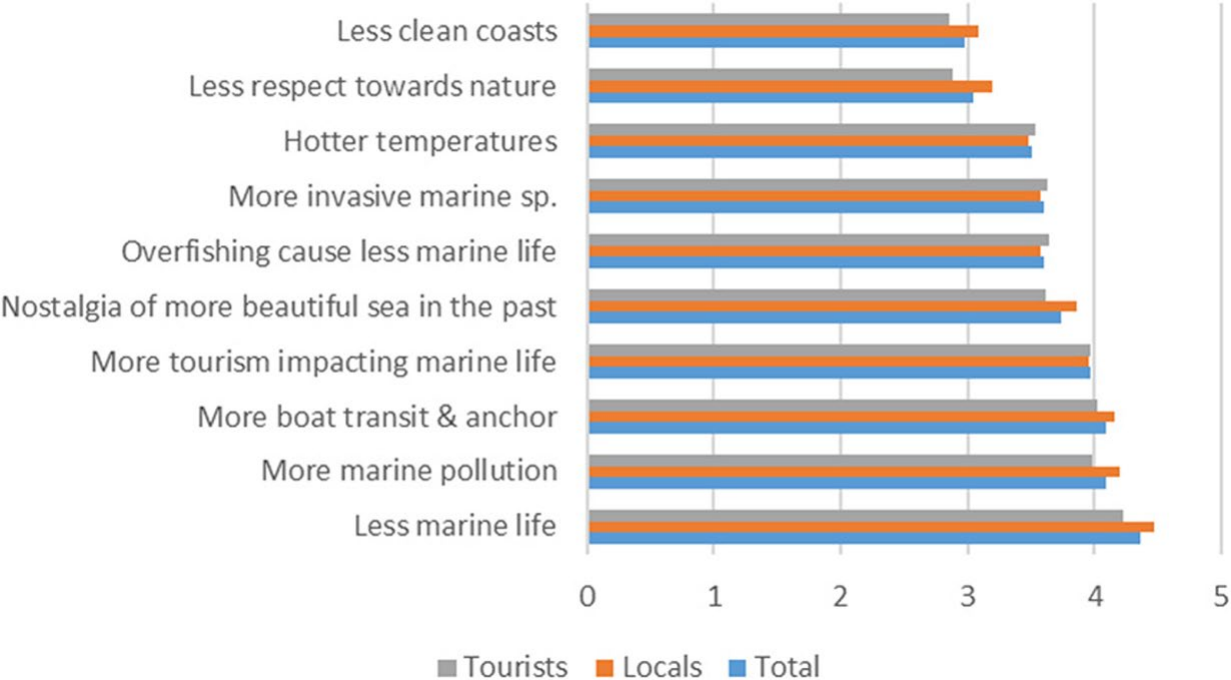
Average score in Likert-scale statements, by locals and tourists (*n* = 427)

The average numerical value of the ten Likert-scale statements was 3.7 (± 0.51), suggesting that, in general, survey participants perceive a deterioration of the environmental status of the CMHs of Cap de Creus. Across reports, the statement that there is “less marine life” now than in the past is the one with the highest average score and the lowest standard deviation (4.36 ± 0.85) (Fig. 3). Three other statements, i.e., “more marine pollution” (4.09 ± 1.04), “more boat transit and anchor” (4.09 ± 1.03), and “more tourism impact” (3.96 ± 1.04), have an average value that would correspond to the category “agree.” Statements with averages below the value that correspond to “agree” include “nostalgia of a more beautiful sea” (3.74 ± 1.1), “overfishing” (3.07 ± 1.03), “more invasive species” (3.60 ± 0.9), and “hotter temperatures” (3.51 ± 1.16). Finally, the statements “less respect towards nature” (3.04 ± 1.19) and “less clean coasts” (2.97 ± 1.19) have average values that correspond to “neither agree nor disagree” (Fig. 3).

Results of the Kruskal–Wallis’s rank-sum test show that, overall, local inhabitants perceive more environmental changes in CMHs than tourists, the difference being statistically significant (3.75 ± 0.52 locals vs. 3.64 ± 0.48 tourists; *p* = 0.009). Indeed, for five of the ten statements in the survey, locals showed stronger agreement with statements about change than tourists did, the difference being statistically significant. Thus, 64.0% of locals, but only 48.3% of tourists “strongly agree” with the statement that there is “less marine life” now than in the past, while only 13.1% of locals, but 21% of tourists, marked that they were neutral or disagreed with the statement (*p* = 0.001). A similar trend is found for the statement refering that there is “more marine pollution” (*p* = 0.005), although in this case tourists more frequently “agree” with the perception of change than locals. Locals displayed more “nostalgia of a more beautiful sea” than tourists, since more locals (35.1%) than tourists (22%; *p* = 0.012) answered that they “strongly agree” with the statement. The same pattern occurred in statements referring to “respect towards nature” (*p* = 0.008) and “less clean coasts” (*p* = 0.045), although for these two statements, tourists selected the option “disagree” more frequently than locals (37.1% and 38% respectively).

The last part of the analysis assesses the differences between locals and tourists considering other sociodemographic characteristics. Results from a Kruskal–Wallis rank-sum test from crossing the variable that define a person as local or tourist with the sociodemographic characteristics used in the analysis suggest that age and affinity/dependence on CMH are the two characteristics that differentiate locals’ and tourists’ responses the most, locals displaying higher levels of affinity/dependence of CMH than tourists, and tourist being generally older than locals (Table 3).

**Table 3:** Statistical significance (*p*-values) from the Kruskal–Wallis rank-sum test from crossing the variable “local/tourist” against six sociodemographic variables, by Likert-scale statement

Statements	Local/tourist × Residence-to-sea distance	Local/tourist × Affinity/dependence	Local/tourist × Education level	Local/tourist × Income	Local/tourist × Age	Local/tourist × Baseline decade
Less marine life	0.02*	0.000**	0.035*	0.069	0.001**	0.001**
More marine pollution	0.008**	0.006**	0.03*	0.155	0.021*	0.125
More boat transit and anchor	0.038*	0.003**	0.31	0.086	0.302	0.149
More tourism impact	0.142	0.003**	0.03*	0.081	0.037*	0.047*
Nostalgia of a more beautiful sea	0.005**	0.001**	0.052	0.155	0.007**	0.099
Overfishing	0.98	0.103	0.642	0.912	0.644	0.603
More invasive species	0.942	0.000**	0.917	0.086	0.236	0.761
Hotter temperatures	0.292	0.965	0.382	0.344	0.013*	0.745
Less respect towards nature	0.071	0.137	0.206	0.069	0.031*	0.19
Less clean coasts	0.128	0.205	0.07	0.004**	0.000**	0.000**
**Average**	0.006**	0.001**	0.096	0.036*	0.001**	0.032*

Significant different values are shown with asterisks: (*) *p* < 0.05, (**) *p* < 0.01. Significant values in bold are the ones that, after a post hoc Dunn test, a significant difference is found between at least one subsample of locals and tourists (see Online Resource 5 for more detail)

We then compared the average of the ten Likert-scale statements across subsamples (i.e., considering the different categories within each variable) to assess which characteristics are more associated with agreement with the deterioration of the CMHs status (i.e., which subsample has an average closer to 5). We found that people who reside in a coastal area show averages closer to 5 than people who live far from them. We found a similar pattern when examining other variables, so people who depend on and/or have a strong affinity with CMHs, people who have low income, and people who are younger than 30 years of age show stronger agreement with the deterioration of the CMHs status than people without these characteristics. The education level and reference baseline categories do not show a clear pattern nor significant differences across samples (Table 4).

**Table 4:**
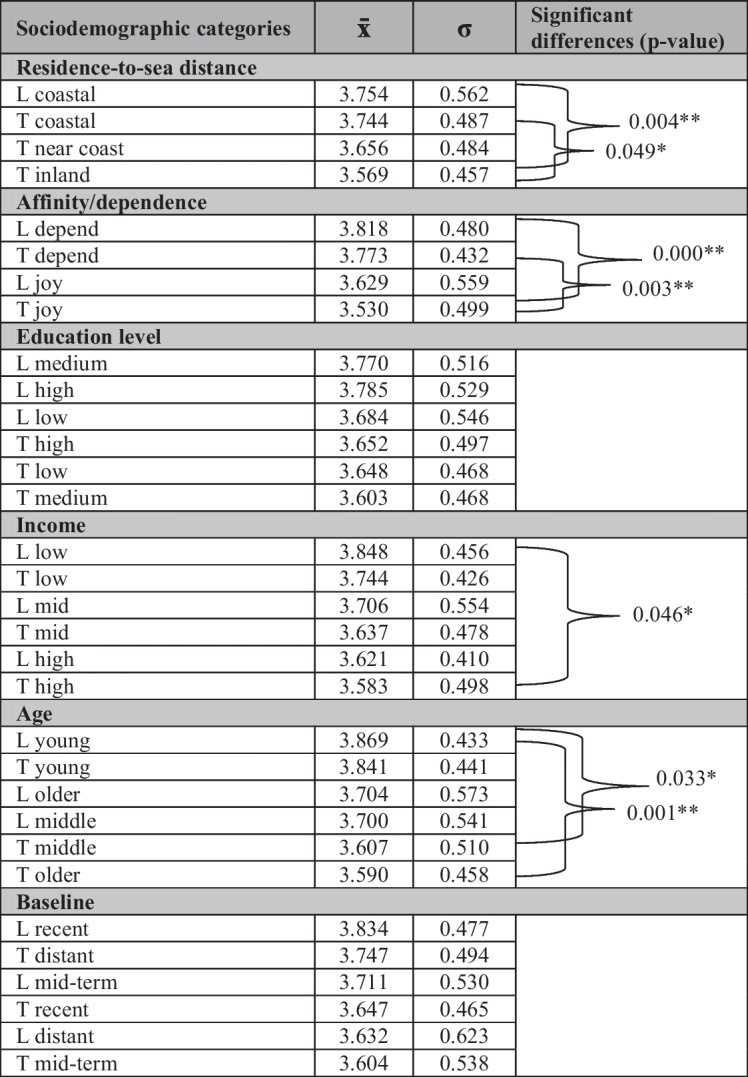
Average answer to ten Likert-scale statements by subsamples of locals (L) and tourists (T) and ordered from highest to lowest value

In the last column, the subcategories that are statistically significant between them are linked with the p-value obtained from the post hoc Dunn test with the Holm method for *p*-values adjustment. (*) *p* < 0.05, (**) *p* < 0.01

The average value for the ten Likert-scale statements calculated for the different subsamples shows that the local respondents most strongly agree with the statements referring to the CMH deterioration. Variables “affinity/dependence” and “age” display the highest statistical differences between the subsamples of locals and tourists, while “education level” has none (Online Resource 6). Locals living near the sea agreed more with the statements that now there is “less marine life” (average: 4.48), “more marine pollution” (4.19), and have “nostalgia of a more beautiful sea” (3.86) than tourists who have their residence inland (4.19, *p* = 0.026; 3.91, *p* = 0.04; and 3.53, *p* = 0.045, respectively). The same pattern is found between locals who are dependent or strongly affine to the CMHs (marine life: 4.6, marine pollution: 4.29, nostalgia: 3.89) compared with tourists who enjoy but do not depend on CMHs (4.1, *p* = 0.000; 3.98, *p* = 0.026, and 3.39, *p* = 0.001 respectively). Locals also display stronger agreement with statements referring to changes on boat transit and anchor (locals: 4.30, tourists: 3.91, *p* = 0.005) and tourism impact (4.07, 3.77, *p* = 0.026). Locals with low-income level display more agreement with the statement that coasts became less clean in recent times, in contrast with tourists with high income (3.41, 2.78, *p* = 0.035). Young locals agree more with the statements that there is now “less respect towards nature” (3.37), “less clean coasts” (3.56), and “more marine pollution” (4.35) than in the past compared to older tourists (2.75, *p* = 0.01; 2.6, *p* = 0.016, and 3.95 *p* = 0.016 respectively). Locals with a distant or mid-term baseline report more agreement with the statement that there is “less marine life” in the present than tourists with a recent baseline (recent tourists: 4.14, distant locals: 4.77; *p* = 0.001, mid-term locals: 4.49; *p* = 0.023; Online Resource 6).

## Discussion

In this work, we identify changes locally reported in Cap de Creus, NE Spain, and assess how locals and tourists perceive environmental changes. Three main findings emerge from our study. First, environmental changes are least reported than economic and social changes. Second, environmental changes are generally reported as negative. And third, local inhabitants generally display higher levels of agreement with statements referring to the deterioration of CMHs than tourists, although differences are mediated by informants’ sociodemographic characteristics. Before we discuss these findings, we present one caveat and two biases of our work that call for caution when interpreting our results.

An important caveat of our work is that our data collection methods only allow to capture some of the changes occurring in the area and neglect the interlinked nature of the reported changes. For example, we did not ask about changes in different ecosystems. Similarly, our coding system forced answers from semi-structured interviews into three dimensions (i.e., economic, social, environmental), ignoring that many changes could be classified in several ways and that there are overlaps across categories. Consequently, our data collection methods are only able to provide a simplified and fragmented view of a complex reality. Additionally, our data might be biased in two different ways. On the one side, as is the case in all research using self-reports, our data can be affected by complacency bias, or informants’ tendency to respond survey questions according to what they think the interviewer wants to hear. Since this bias might affect all informants on the same level, we do not expect that it will affect the estimations across different groups of respondents. On the other side, our results might also be affected by omitted variable biases. Our starting point is that environmental perception is shaped by many factors, but we could include only some of them in our analysis. Variables not included in our analysis (e.g., profession, knowledge of other coastal areas) might also influence perceptions of change and omitting them might have biased our results in unknown magnitude and direction.

Considering these caveats, the first important finding of this work is that environmental changes are least reported than economic and social changes. The dominance of reports of economic changes in semi-structured interviews probably reflects the dramatic economic changes occurring in the study area over the last decades. As mentioned, tourism has become the main source of income in this coastal region, with a predominance of seasonal tourism (Gómez et al. 2006). During interviews, informants commented how tourism has changed the economic, social, and environmental dynamics of the area, with reports of impacts ranging from the impression that tourists now stay less time and spend less money than before to reports of tourism impacts on marine life. Results on the multidimensional impacts of tourism are in line with results reported in other coastal areas, where people have reported the environmental (Garcés-Ordóñez et al. 2020; Grelaud & Ziveri 2020; Silva et al. 2018), but also the social impacts derived from tourism (Ap 1992; Perdue et al. 1990; Smith et al. 2023). Indeed, a recent review of the literature of the impacts of tourism on coastal areas points to a general scientific agreement that tourism results in positive economic impacts, but negative social and environmental impacts. Moreover, positive economic impacts benefit only a few, particularly with the increasing domination of large corporations in the turistic sector (Smith et al. 2023).

The second main finding of this work refers to the clear dominance of reports of negative over positive changes. In line with general trends found in the literature (Smith et al. 2023), our survey results indicate overall agreement with the deterioration of the environmental quality of the area. In particular, we found a strong agreement that there is now “less marine life.” The finding that local people perceive a decrease of marine biodiversity is not new, as previous work documents a generalized public perception of the environmental deterioration of marine systems (Jefferson et al. 2014; Tonin & Lucaroni 2017) and describes drivers of marine habitat change (Abecasis et al. 2013; Arulnayagam 2020; Pita et al. 2013; Tran et al. 2002). Moreover, beyond public perception, biodiversity decline is well reported in Mediterranean CMHs, a deterioration that affects different habitats, such as Mediterranean seagrass (*Posidonia oceanica*) (Marbà et al. 2014) and red coral (Bavestrello et al. 2015), but also the abundance and size of commercial marine species or large predators (Ferretti et al. 2008; Lotze et al. 2011; Montefalcone et al. 2018). Furthermore, and despite the specific role of the MPA in the recovery of some key species and habitats (Mallo et al. 2019), biodiversity decline and environmental quality worsening have also been documented in Cap de Creus. For example, previous work has documented that local artisanal fisherfolk notice a decrease in vulnerable species landings (Gómez et al. 2006) and catches weight and size (Font & Lloret 2011), and that the area also suffers from other pressures contributing to biodiversity loss like litter, pollution, and climate change (Purroy et al. 2014; Sardá et al. 2012). In other words, the local perception of a decline in the biodiversity of the area generally overlaps with findings from studies in the region relying on instrumental data.

The third important finding of this work is that local inhabitants generally show stronger agreement with negative environmental changes than tourists. We argue that the main explanation of this difference rests in the deeper knowledge of the area that locals have, which allows them to perceive more changes or changes that are only visible over long periods of time. Indeed, the contribution of local knowledge to assessing environmental change is a well-researched topic (see Azzurro et al. 2019; Reyes-García et al. 2024; Weatherhead et al. 2010). Differences among locals and tourists perceptions could also be amplified by the fact that locals usually care more about their surrounding environment than tourists, something well reported in the literature when assessing the “Not In My Back Yard” (NIMBY) and “Locally Unwanted Land Use” (LULU) phenomena (Schively 2007).

Importantly, we found that—beyond their status as local or tourist—there are sociodemographic differences between the two subsamples that might help explain the differences found. Respondents who lived close to the sea and who were more affine with or dependent on the CMH exhibited stronger agreement with statements of negative environmental change that respondents without these characteristics. In fact, these characteristics (e.g., residence-to-sea proximity, affinity/dependence of the CMHs) are proxies for connectedness to nature, which has been shown to shape environmental change perceptions (García-del-Amo et al. 2023; Mayer & Frantz 2004). For example, fishers or scuba divers with a strong affinity/dependence of the CMH may agree with statements of negative change because they observe the deterioration of the habitat through their constant interactions with nature. More connections with nature often result in higher levels of concern for it (Mayer & Frantz 2004; Vining et al. 2008). The same argument might, indeed, apply to the finding that locals with low-income levels display more agreement with statements regarding coastal deterioration than richer tourists. In fact, an important part of low-income jobs in our study context are primary activities, linked to the coastal setting and that require direct contact with nature. So, in our study context, a low-income level might actually be a proxy for dependency on nature, a phenomenon that has also been reported in other areas of rural Spain (García-del-Amo et al. 2023). Finally, we also found that, compared to older tourists, younger locals agree more with the statements that there is now less respect towards nature, and that the coast is less clean and more polluted than in the past. The finding is surprising, as younger respondents might be comparing to a closer baseline, for which result might just be reflecting a generational effect. Younger participants might have been exposed to more information regarding environmental (Jacquet & Pauly 2007), which might condition the way they responded to our survey.

## Conclusion

This study adds to previous works analyzing perceptions of environmental change, focusing on perceptions of change in coastal marine environments. We found that, although environmental changes do not seem to be local users’ main concern, as they focused on the report of economic changes, there is a general perception that the local marine habitat is deteriorating, a perception consistent with scientific assessments of the health status of the area. Moreover, we also found that local inhabitants, more than tourists, perceive a stronger decline in coastal marine environment status. The reported negative environmental trend is particularly alarming since the study area has been a MPA for more than 20 years. The increasing disconnection of people with nature is a main underlying cause of nature decline worldwide (Díaz et al. 2019; Pascual et al. 2023), and we found that people with stronger connections to nature have stronger perceptions of nature decline. Increasing people’s connection with nature is needed to increase a societal environmental concern which might potentially result in pro-environmental behaviors and actions.

## Supplementary Information

Below is the link to the electronic supplementary material.

Supplementary file1 Online Resource 1. Samples in semi-structured interviews and survey, by municipality. Tables include information on the sample participating, the total number of people approached, and the reasons for declining participation. (PDF 330 KB)

Supplementary file2 Online Resource 2. Survey. The table in the last page includes the original statements in the survey, an indication of the ones that were reversed for data analysis, and the correspondence with abbreviations used in the article. (PDF 333 KB)

Supplementary file3 Online Resource 3. Changes reported in semi-structured interviews (n = 38). ‘Types of changes’ refers to items in the list of changes reported; “Frequency of changes” refers to the total number of mentions during semi-structured interviews. (PDF 16 KB)

Supplementary file4 Online Resource 4. Raw data. Country: Abbreviated by ISO alpha-3 coding. See description of the variables in Table 1. (PDF 1032 KB)

Supplementary file5 Online Resource 5. Distribution of responses to 10 Likert scale statements. (PNG 55 KB)

Supplementary file6 Online Resource 6. Spider diagrams comparing average values of responses to Likert scale statements, by subsamples differentiating between locals and tourists with different characteristics. Concentric polygons represent the axis of the average values, where 1 = “strongly disagree”, 2 = “disagree”, 3 = “neither agree nor disagree”, 4 = “agree”, and 5 = “strongly agree”. Each spike represents an abbreviation of each statement in the Likert table (see Online Resource 2). Statements highlighted in yellow have averages with statistically significant differences between local and tourist subsamples in a Kruskal-Wallis rank sum test (Table 2). In italics the subsamples for which we found statistically significant differences in a post-hoc Dunn test (p-value inside parenthesis; (*) *p* < 0.05, (**) *p* < 0.01). (PDF 517 KB)

## Data Availability

The data analyzed during the current study are available as Supplementary Material 4.
