# Supplementary material for: Coastal marine habitats deterioration according to users’ perception: the case of Cap de Creus Marine Protected Area (NE Spain)
Source: Reg Environ Change. 2024 Oct 10;24(4):155. doi: 10.1007/s10113-024-02322-4 (PMC11467071; doi:10.1007/s10113-024-02322-4)
Supplement: Supplementary file 1 — Supplementary file1 Online Resource 1. Samples in semi-structured interviews and survey, by municipality. Tables include information on the sample participating, the total number of people approached, and the reasons for declining participation. (PDF 330 KB) [file 10113_2024_2322_MOESM1_ESM.pdf]

Online Resource 1

**Semi-structured interviews**					**Motive of no answer**				
**Interviewed (Potential interviewees)**	**Roses**	**Cadaqués**	**Port de la Selva**	**Total**	**Lack of time**	**Unwill**	**< 10 years**	**N/A**	**Total**
Fresh seafood restaurants	5 (12)	5 (14)	4 (5)	14 (31)	8	4	3	2	17
Coastal hotels and hostels	10 (30)	8 (17)	3 (4)	21 (51)	6	7	6	11	30
Diving centres	1 (5)	3 (4)	1 (2)	5 (11)	2	0	0	4	6
Total	16 (47)	16 (35)	8 (11)	40 (93)	16	11	9	17	53

**Survey**	**Total**	**Female**	**Male**	**Motive of no answer**						
**Respondants**	427	245	182	**Lack of time**			**Unwill**	**Language barrier**	**< 10 years**	**Other**
**Non respondants**	800	449	351	178			359	36	197	30
				Due to work	Due to appointment	Unknown				
				75	41	62				

Semi-structured interviews: The establishments that closed and the new ones that opened after the elaboration of the potential list of interviewees (winter 2017) were not counted nor considered for interview. The restaurants that in the moment of the interview said that they did not serve fresh seafood were not counted in the list as well. The owners or managers of more than one type of establishment (e.g. owner of two hotels) where counted only as one in the list of potential interviewees; this happened six times. The people that own two different establishments (e.g. and hostel and a restaurant) where counted as two despite a single interview was realized; these happened two times. We excluded people with less than ten years working or diving in the area ("< 10 years" category). For the particular case of fresh seafood restaurants in Roses, in order to avoid a long and time-consuming list of restaurants, we decided to include in the list of potential interviewees only the fresh seafood restaurants that appeared in the "Suquet de Peix" (Fish Suquet) catalogue handed to us by the office of tourism in winter 2017. An actualized version of such catalogue can be consulted online (http://en.visit.roses.cat/plan/restaurants/).

Survey: Before answering the questionnaire, the interviewer introduced itself, the institution that was working from (ICTA-UAB) and its position (doctoral student, fieldwork assistant, researcher) and explained the project and the objective of this questionnaire to the interviewees. We also reminded them that they will remain anonymous and that we disposed of a text-version of the informed consent expressed beforehand orally. The researcher helped to clarify any doubt the respondent had in a neutral way, i.e., such help never implied conditioning the answer of the respondent. We had translated the survey to the four most common languages spoken by residents and tourists in the area: Spanish, Catalan, English, and French. We counted the people who did not understand any of those languages as non-respondents, inside the category of "Language barrier". We excluded people that visited or lived in the area for less than ten years "< 10 years".

Coastal marine habitats deterioration. Perception of Cap de Creus Marine Protected Area (NE Spain) users. Regional Environmental Change. Miguel Mallo, Patrizia Ziveri, Sergio Rossi, Victoria Reyes-García. Corresponding authors: Miguel Mallo (miguelmallo91@gmail.com), Patrizia Ziveri (Patrizia.ziveri@uab.cat). Institut de Ciència i Tecnologia (ICTA). Universitat Autònoma de Barcelona (UAB), Bellaterra, Barcelona, Spain.

ID	Interview date	Municipality	Ocupation	Gender	Born Year	Start activity
1	19/12/2017	Port de la Selva	Restaurant	Male	1948	1963
2	19/12/2017	Cadaqués	Restaurant	Male	1973	2002
3	20/12/2017	Roses	Restaurant	Male	1963	2001
4	20/12/2017	Roses	Restaurant	Male	1962	1999
5	20/12/2017	Roses	Hostal	Female	1958	1973
6	20/12/2017	Roses	Hostal	Female	1990	2002
7	20/12/2017	Roses	Restaurant	Male	1946	1955
8	21/12/2017	Roses	Restaurant	Male	1982	1997
9	22/12/2017	Roses	Hostal	Male	1980	1993
10	20/7/2018	Roses	Hostal	Female	1969	1983
11	20/7/2018	Roses	Hostal	Male	1945	1963
12	21/7/2018	Roses	SD	Male		
13	21/7/2018	Roses	SD	Male	1968	1982
14	21/7/2018	Port de la Selva	Restaurant	Male	1979	1992
15	21/7/2018	Port de la Selva	Restaurant	Female	1967	2000
17	21/7/2018	Port de la Selva	Hostal & Restaurant	non binary	1970	1985
18	22/7/2018	Roses	Hostal	Male	1994	2009
19	23/7/2018	Roses	Restaurant	Female	1967	1975
20	23/7/2018	Cadaqués	SD	Male	1973	1998
21	23/7/2018	Cadaqués	Hotel	Female	1974	1994
22	23/7/2018	Cadaqués	Hostal	Female	1966	1982
23	24/7/2018	Roses	Hotel	Male	1974	1990
24	24/7/2018	Roses	Hotel	Female	1976	2006
25	24/7/2018	Roses	Hotel	Female	1972	1988
27	24/7/2018	Cadaqués	Restaurant	Male	1965	1978
28	24/7/2018	Cadaqués	SD	Male	1979	2010
29	25/7/2018	Cadaqués	Hostal	Male	1970	1984
30	25/7/2018	Cadaqués	Restaurant	Male	1957	1967
31	25/7/2018	Cadaqués	Hotel	Male	1967	1983
32	25/7/2018	Port de la Selva	SD	Male	1962	1994
33	25/7/2018	Port de la Selva	Hostal	Male	1947	1968
34	26/7/2018	Roses	Hotel	Male	1959	1981
35	26/7/2018	Cadaqués	SD	Male	1966	1989
36	27/7/2018	Cadaqués	Restaurant	Male	1975	2001
37	27/7/2018	Cadaqués	Hotel	Female	1952	1968
38	27/7/2018	Cadaqués	Hostal	Female	1971	1984
39	27/7/2018	Cadaqués	Restaurant	Female	1988	2002
40	27/7/2018	Cadaqués	Hotel	Male	1943	1999

FGD POTENTIAL LOCAL EXPERTS

MPA 1		MPA Technicians	Female	MPA director	Victòria Riera: v
MPA 2		MPA Technicians	Male		Gerard
SD 1	Port de la Selva	Recreative scuba diver	Female		Júlia
SD 2	Port de la Selva	Recreative scuba diver	Male		Josep Anton: su
SD 4	Roses	Recreative scuba diver		Enviar e.mail: inf	Álvaro López: n

ictoria.riera@gencat.cat / 972193191

6,07E+08	972 387 000
6,07E+08	972 387 000

nanager: no estava dispost
