# Supplementary material for: Coastal marine habitats deterioration according to users’ perception: the case of Cap de Creus Marine Protected Area (NE Spain)
Source: Reg Environ Change. 2024 Oct 10;24(4):155. doi: 10.1007/s10113-024-02322-4 (PMC11467071; doi:10.1007/s10113-024-02322-4)
Supplement: Supplementary file 2 — Supplementary file2 Online Resource 2. Survey. The table in the last page includes the original statements in the survey, an indication of the ones that were reversed for data analysis, and the correspondence with abbreviations used in the article. (PDF 333 KB) [file 10113_2024_2322_MOESM2_ESM.pdf]

Online Resource 2

Date (dd/mm/yy):_____/_____/_____ **Code#:**___________ (leave this blank) **ENGLISH**

Gender: Female Male Other:_______________

Town/city of residence:____________________ **Country:** ___________________________

Where are you hosted? (answer only if you do not live here)**:** Port de la Selva Roses Cadaqués

How often do you visit this area? (select one box only)

- ☐ I live here all year round → Since what decade?:__________ or Since birth
- ☐ Once every year → What was the decade of your first visit? _______________
- ☐ Frequently → What was the decade of your first visit? _______________
- ☐ Infrequently → What was the decade of your first visit? _______________
- ☐ This is my first time

⚠ ***If you visited this area <u>for the first time</u> you do not have to answer this table.***

How much do you agree with the following statements? Always refer your answers to this area (Cap de Creus). Note that some sentences are comprised of more than one statement; your answer must take into account the full sentence and not only one part. When the statements mention "in the past" refer to your first visit to the area or, if you are born here, since your childhood.

Statement	N/A	Strongly disagree	Disagree	Neither agree nor disagree	Agree	Strongly agree
In comparison with today, in the past there were less long-lasting hot temperatures. In other words, summers were shorter.	0	1	2	3	4	5
In comparison with today, in the past more fishes and other marine species were visible in the sea.	0	1	2	3	4	5
In comparison with the past, people are today more aware of the importance of respecting the natural areas they are visiting (for example, not littering, not stepping on marine life or collecting plants or animal souvenirs…).	0	1	2	3	4	5
In comparison with the past, today the coast (including beaches) remains cleaner and better taken care of.	0	1	2	3	4	5
In the past the sea was more polluted than today.	0	1	2	3	4	5
In comparison with the past, today there exists more boat transit and boat anchoring; this increases the negative impacts on marine life.	0	1	2	3	4	5
In comparison with the past, today there are more invasive marine species that come from outside and negatively affect the local marine life.	0	1	2	3	4	5
Overfishing is the main cause of marine life decrease.	0	1	2	3	4	5
People feel nostalgia about how beautiful the sea used to be and complain that the younger generation never saw it like they did.	0	1	2	3	4	5
This area receives increasingly tourism, which has a growing negative impact on marine life.	0	1	2	3	4	5

To what extent do you think you depend on the marine environment in your daily life, either for your work and/or income or for your leisure activities? (select one box only)

For example, you might be strongly dependent if your work is highly based on the sea, like fishermen, whale watching, lifeguard… or your hobby, like surfing, swimming, diving… or simply the need of going to visit the coastline.

- ☐ Strongly. I need it frequently
- ☐ I need it once in a while, I cannot spend too much time without it
- ☐ I enjoy it, but I do not depend on it
- ☐ Indifferent. It has nothing to do with me
- ☐ I avoid it

Age group: (select one box only)

- ☐ 18 – 29
- ☐ 30 – 39
- ☐ 40 – 49
- ☐ 50 – 59
- ☐ 60 – 69
- ☐ 70 – 79
- ☐ 80 – 89
- ☐ ≥ 90

What is the highest level of education that you have completed?: (select one box only)

- ☐ No schooling completed
- ☐ Primary school
- ☐ Middle school
- ☐ High school
- ☐ Professional degree/technical school training
- ☐ University
- ☐ Master, post-graduate
- ☐ Doctorate/PhD

What is your profession? ____

Choose one of the following that best indicate your average yearly income. If you are retired think in your income when you were working:

(select only one box and the one in your currency)

**Euros (€)**	**Dollars ($)**	**Pound sterling (£)**
☐ No income/unemployed	☐ No income/unemployed	☐ No income/unemployed
☐ < 14,000 €	☐ < 15,000 $	☐ < 12,000 £
☐ 14,000 – 35,000 €	☐ 15,000 – 40,000 $	☐ 12,000 – 30,000 £
☐ 35,000 – 56,000 €	☐ 40,000 – 60,000 $	☐ 30,000 – 50,000 £
☐ > 56,000 €	☐ > 60,000 $	☐ > 50,000 £

[*Not part of the questionnaire*]

Original statements used in the questionnaire, the ones that we twisted their answers' value for the data analysis, and the two different abbreviations of the statements used inside the article.

Original Statement	Twisted order?	Abbreviation	Abbreviation 2
In comparison with today, in the past there were less long-lasting hot temperatures. In other words, summers were shorter.		Hotter temperatures	Hot Tº
In comparison with today, in the past more fishes and other marine species were visible in the sea		Less marine life	Marine life
In comparison with the past, people are today more aware of the importance of respecting the natural areas they are visiting (for example, not littering, not stepping on marine life or collecting plants or animal souvenirs…)	Yes	Less respect towards nature	Respect towards nature
In comparison with the past, today the coast (including beaches) remains cleaner and better taken care of	Yes	Less clean coasts	Clean coasts
In the past the sea was more polluted than today	Yes	More marine pollution	Marine pollution
In comparison with the past, today there exists more boat transit and boat anchoring; this increases the negative impacts on marine life		More boat transit and anchor	Boat transit and anchor
In comparison with the past, today there are more invasive marine species that come from outside and negatively affect the local marine life		More invasive species	Invasive species
Overfishing is the main cause of marine life decrease		Overfishing	Overfish
People feel nostalgia about how beautiful the sea used to be and complain that the younger generation never saw it like they did		Nostalgia of a more beautiful sea	Nostalgia
This area receives increasingly tourism, which has a growing negative impact on marine life		More tourism impact	Tourism

 Miguel Mallo, Patrizia Ziveri, Sergio Rossi, Victoria Reyes-García. Corresponding authors: Miguel Mallo (miguelmallo91@gmail.com), Patrizia Ziveri (Patrizia.ziveri@uab.cat). Institut de Ciència i Tecnologia (ICTA). Universitat Autònoma de Barcelona (UAB), Bellaterra, Barcelona, Spain.
