# Supplementary material for: Coastal marine habitats deterioration according to users’ perception: the case of Cap de Creus Marine Protected Area (NE Spain)
Source: Reg Environ Change. 2024 Oct 10;24(4):155. doi: 10.1007/s10113-024-02322-4 (PMC11467071; doi:10.1007/s10113-024-02322-4)
Supplement: Supplementary file 3 — Supplementary file3 Online Resource 3. Changes reported in semi-structured interviews (n = 38). ‘Types of changes’ refers to items in the list of changes reported; “Frequency of changes” refers to the total number of mentions during semi-structured interviews. (PDF 16 KB) [file 10113_2024_2322_MOESM3_ESM.pdf]

Online Resource 3

	Total			Economic			Social			Environmental		
	Both	(+)	(-)	Both	(+)	(-)	Both	(+)	(-)	Both	(+)	(-)
**Types of change**												
n	63	24	39	26	11	15	19	7	12	18	6	12
% respect total	100	38	62	41	17	24	30	11	19	29	10	19
% respect (+)		100			46			29			25	
% respect (-)			100			38			31			31
**Frequency of changes**												
n	365	93	272	171	54	117	106	21	85	88	18	70
Average/interview	9.6	2.4	7.2	4.5	1.4	3.1	2.8	0.6	2.2	2.3	0.5	1.8
% respect total	100	25	75	47	15	32	29	6	23	24	5	19
% respect (+)		100			58			23			19	
% respect (-)			100			43			31			26

Coastal marine habitats deterioration. Perception of Cap de Creus Marine Protected Area (NE Spain) users. Regional Environmental Change. Miguel Mallo, Patrizia Ziveri, Sergio Rossi, Victoria Reyes-García. Corresponding authors: Miguel Mallo (miguelmallo91@gmail.com), Patrizia Ziveri (Patrizia.ziveri@uab.cat). Institut de Ciència i Tecnologia (ICTA). Universitat Autònoma de Barcelona (UAB), Bellaterra, Barcelona, Spain.
