# Supplementary material for: Coastal marine habitats deterioration according to users’ perception: the case of Cap de Creus Marine Protected Area (NE Spain)
Source: Reg Environ Change. 2024 Oct 10;24(4):155. doi: 10.1007/s10113-024-02322-4 (PMC11467071; doi:10.1007/s10113-024-02322-4)
Supplement: Supplementary file 4 — Supplementary file4 Online Resource 4. Raw data. Country: Abbreviated by ISO alpha-3 coding. See description of the variables in Table 1. (PDF 1032 KB) [file 10113_2024_2322_MOESM4_ESM.pdf]

Online Resource 4

				Sociodemographic parameters								Statements (See SM 2 for the entire statement)										
Code	Place of interview	Gender	Country	Local / Tourist	Residence-to-sea distance	Frequency & Antiquity	Marine affinity/ dependence	Age group	Education level	Income	Baseline decade	Hot T°	Marine life	Respect towards nature	Clean coasts	Marine pollution	Boat transit & anchor	Invasive species	Overfish	Nostalgia	Tourism	Average
SC14	Cadaqués	Female	FRA	Tourist	Inland		Joy	70-79	No Sch.	14-35		4	3	2	2	2	4	3	5	3	4	3.2
SC11	Cadaqués	Male	ESP	Tourist	Near coast	Frequent	Joy	50-59	High Sch.	14-35	2000s	3	5	5	5	5	5	3	4	4	4	4.3
SC07	Cadaqués	Male	NLD	Tourist	Coastal	Frequent	Joy	18-29	High Sch.	14-35	2000s	4	5	2	3	5	5	3	3	5	5	4.0
SC06	Cadaqués	Male	NLD	Tourist	Coastal	Frequent	Joy	18-29	Master	35-56	1990s	4	3	3	2	2	3	3	3	4	3	3.0
SC05	Cadaqués	Male	FRA	Tourist	Near coast	Frequent	Joy	50-59	University	35-56	1980s	1	5	4	1	5	5	4	3	4	4	3.6
SC02	Cadaqués	Female	ESP	Tourist	Near coast	Frequent	Joy	80-89	No Sch.	14-35	1980s	5	5	5	1	5	2	4	4	5	3	3.9
SC03	Cadaqués	Female	ESP	Tourist	Coastal	Frequent	Joy	60-69	Middle Sch.	14-35	1980s	5	5	5	4	5	5	4	4	5	5	4.7
SC01	Cadaqués	Female	ESP	Tourist	Coastal	Frequent	Joy	70-79	Primary Sch.	14-35	1970s	5	5	5	1	5	2	4	4	5	3	3.9
SC04	Cadaqués	Female	ESP	Tourist	Near coast		Strong	40-49	Prof./Tech.	14-35		5	5	4	4	4	4	4	4	2	4	4.0
AR11	Roses	Female	FRA	Tourist	Inland		Joy	50-59	Prof./Tech.	14-35		2	5	1	4	3	4	4	4	5	4	3.6
AR10	Roses	Male	ROU	Tourist	Inland	Frequent	Joy	18-29	High Sch.	14-35	2000s	4	5	2	4	5	3	1	3	1	3	3.1
AR06	Roses	Male	ESP	Local	Coastal	Native	Joy	40-49	High Sch.	14-35	1980s	3	5	3	3	3	4	3	3	5	5	3.7
AR05	Roses	Female	ESP	Local	Coastal	Native	Strong	40-49	University	<14	1980s		5	5	4	4	5	5	5	5	5	4.8
AR04	Roses	Female	ESP	Local	Coastal	Native	Joy	30-39	Prof./Tech.	14-35	1990s	2	4	4	4	4	4	3	4	2	4	3.5
AR01	Roses	Male	BEL	Tourist	Inland	Frequent	Joy	50-59	University		1990s	4	5	2	2	2	3	3	5	3	4	3.3
WP07	Port Selva	Male	ESP	Local	Coastal	Native	Joy	70-79	High Sch.		1950s	4	5	3	4	4	4	5	5	4	4	4.2
WP08	Port Selva	Male	ESP	Local	Coastal	Native	Strong	70-79	Middle Sch.		1950s	5	5	3	2	5	3	5	1	1	1	3.1
WP11	Port Selva	Male	LUX	Tourist	Inland	Frequent	Joy	50-59	University	>56	1970s	4	5	3	4	5	4	4	3	5	3	4.0
WP12	Port Selva	Female	LUX	Tourist	Inland	Frequent	Joy	50-59	Prof./Tech.	>56	2000s	4	5	3	2	4	3	3	5	4	4	3.7
WP13	Port Selva	Female	FRA	Tourist	Inland	Frequent	Joy	40-49	PhD	>56	1970s	3	5	3	2	5	5	5	3	3	4	3.8
WP14	Port Selva	Male	FRA	Tourist	Inland	Frequent	Joy	40-49	Master	>56	2000s	3	5	3	3	5	4	4	3	4	4	3.8
WP15	Port Selva	Male	FRA	Tourist		Frequent	Need	50-59	Master	35-56	2000s	3	4	4	2	4	4	2	5	5	4	3.7
WP16	Port Selva	Female	FRA	Tourist		Frequent	Joy	30-39	Master	35-56	1990s	4	3	2	2	3	3	3	2	2	3	2.7
WP17	Port Selva	Female	ESP	Tourist	Coastal	Frequent	Strong	>90	Master		1930s	5	5	4	3	5	5	5	4	5	5	4.6
WP18	Port Selva	Female	ESP	Tourist	Near coast	Frequent	Need	60-69	Primary Sch.		1970s	2	4	2	2	5	5	4	4	3	2	3.3
WP19	Port Selva	Female	ESP	Local	Coastal	Native	Strong	18-29	Middle Sch.	<14	2000s	4	5	5	3	5	3	4	5	5	5	4.4
AP14	Port Selva	Male	AND	Tourist	Inland		Need	60-69	University	>56		2	5	1	3	5	5	5	2	5	2	3.5
AP15	Port Selva	Female	DEU	Tourist		Frequent	Joy	60-69	High Sch.	14-35	1980s	3	3	2	2	3	3	3	3	4	3	2.9
AP16	Port Selva	Female	CHE	Tourist	Inland	Frequent	Joy	50-59	University	>56	1990s	4	4	2	2	4	3	3	4	3	2	3.1
AP17	Port Selva	Female	FRA	Tourist	Inland	Frequent	Joy	50-59	University	14-35	1970s	2	5	2	2	4	2	3	4	4	4	3.2
AP18	Port Selva	Male	ESP	Tourist	Coastal	Frequent	Avoid	70-79	Primary Sch.	<14	1940s	4	5	1	1	3	5	3	1	4	5	3.2
AP20	Port Selva	Female	ESP	Local	Coastal	Native	Joy	60-69	Middle Sch.	14-35	1960s	5	5	1	1	3	2	1	2	4	4	2.8
AP22	Port Selva	Female	ESP	Tourist	Coastal	Frequent	Joy	30-39	Prof./Tech.		2000s	4	3	3	4	2	3	4	3	3	4	3.3
AP24	Port Selva	Female	FRA	Tourist	Inland	Frequent	Joy	18-29	Middle Sch.	No income	2000s	4	4	1	3	4	3	2	5	3	3	3.2
AP25	Port Selva	Female	ESP	Local	Coastal	Native	Strong	18-29	High Sch.	<14	2000s	5	5	4	4	5	5	3	5	5	5	4.6
AP26	Port Selva	Male	ESP	Local	Coastal	Native	Need	50-59	Prof./Tech.	14-35	1970s	4	5	3	2	4	4	3	3	4	3	3.5
SP67	Port Selva	Male	BEL	Tourist	Inland	Frequent	Need	60-69	High Sch.	14-35	1960s	3	4	4	4	4	4	3	2	2	3	3.3
SP51	Port Selva	Male	ESP	Local	Coastal	Native	Joy	80-89	Primary Sch.	14-35	1940s	5	5	5	1	4	3	4	3	5	3	3.8
SP50	Port Selva	Male	ESP	Local	Coastal	Native	Strong	18-29	Prof./Tech.	<14	2000s	4	5	2	2	3	5	3	3	5	5	3.7
SP52	Port Selva	Female	ESP	Tourist	Coastal	Infrequent	Joy	18-29	Master	<14	2000s	3	4	3	3	3	4	3	2	1	4	3.0
SP55	Port Selva	Male	ESP	Local	Coastal	Native	Need	80-89	Middle Sch.	14-35	1940s	4	5	4	2	4	4	4	4	4	4	3.9
SP61	Port Selva	Female	ESP	Tourist	Near coast	Frequent	Need	18-29	High Sch.	No income	2000s	5	3	4	3	5	5	5	3	4	5	4.2
SP66b	Port Selva	Male	ESP	Local	Coastal	Native	Joy	18-29	High Sch.	<14	2000s	3	4	5	4	5	4	4	3	3	2	3.7
MR23	Roses	Female	ESP	Local	Coastal	Native	Strong	50-59	High Sch.	<14	1970s	3	4	4	4	4	4	3	4	4	5	3.9
MR28	Roses	Female	ESP	Local	Coastal	Native	Need	18-29	University	<14	2000s	4	4	4	5	5	4	4	3	4	5	4.2
MR29	Roses	Female	ESP	Local	Coastal	Native	Strong	18-29	High Sch.	No income	2000s	4	4	3	3	4	5	3	2	4	2	3.4
MR32	Roses	Female	ESP	Local	Coastal	Native	Need	18-29	Middle Sch.	<14	2000s	4	5	4	5	5	5	2	5	4	5	4.4
MR33	Roses	Male	ESP	Local	Coastal	Native	Need	30-39	University	14-35	1990s	3	5	1	1	2	4	3	3	1	2	2.5
SR27	Roses	Female	ESP	Local	Coastal	Native	Strong	40-49	Prof./Tech.	14-35	1980s	2	5	3	2	4	4	3	5	5	5	3.8
SR28	Roses	Female	ESP	Local	Coastal	Native	Need	40-49	Middle Sch.	14-35	1980s	3	5	3	3	4	4	4	5	5	4	4.0
SR30	Roses	Male	ESP	Tourist	Near coast	Frequent	Joy	40-49	University	35-56	1980s	1	5	1	1	4	5	3	5	2	4	3.1
SR31	Roses	Male	FRA	Tourist	Inland	Frequent	Joy	60-69	Middle Sch.	<14	2000s	2	5	2	3	5	4	2	5	3	3	3.4
SR33	Roses	Female	FRA	Tourist	Inland	Frequent	Need	50-59	Middle Sch.	<14	1990s	3	5	3	2	4	5	3	5	5	5	4.0
SR36	Roses	Male	FRA	Tourist	Inland	Frequent	Indifferent	60-69	Middle Sch.	14-35	1990s	5	4	3	3	4	4	4	2	5	4	3.8
SR37	Roses	Female	ESP	Tourist	Inland	Infrequent	Joy	60-69	Primary Sch.	14-35	1990s	5	4	2	2	3	4	4	4	4	4	3.6
SR38	Roses	Female	ESP	Local	Coastal	Native	Strong	18-29	University	<14	2000s	4	3	4	2	5	4	4	2	3	3	3.4
SR39	Roses	Female	ESP	Local	Coastal	Non-native	Need	18-29	Middle Sch.		1990s	2	4	1	2	3	4	3	3	3	4	2.9
SR40	Roses	Female	ESP	Local	Coastal	Native	Joy	18-29	University	<14	2000s	5	5	4	4	5	4	2	2	5	5	4.1
SR41	Roses	Female	ESP	Local	Coastal	Native	Strong	50-59	Prof./Tech.	<14	1970s	5	5	3	3	3	2	4	3	4	3	3.5
SR42	Roses	Female	ESP	Local	Coastal	Native	Joy	18-29	University	<14	2000s	3	5	2	5	3	5	3	4	5	1	3.6
SR43	Roses	Female	CAN	Tourist	Inland	Frequent	Need	50-59	Prof./Tech.	14-35	2000s	3	4	3	2	4	3	4	4	2	2	3.1
SR44	Roses	Male	CAN	Tourist	Inland	Frequent	Joy	50-59	Middle Sch.	35-56	2000s	4	3	1	3	5	3	4	5	3	1	3.2
SR45	Roses	Male	ESP	Tourist	Inland	Frequent	Joy	30-39	University	14-35	2000s	3	3	5	5	5	1	3	3	1	1	3.0
SR46	Roses	Female	ESP	Tourist	Inland	Infrequent	Joy	30-39	University	35-56	1980s	4	5	3	3	5	5	4	4	4	4	4.1
SR47	Roses	Female	ESP	Tourist	Near coast	Frequent	Joy	60-69	Primary Sch.	<14	1980s	5	3	4	1	5	5	5	2	4	5	3.9
SR48	Roses	Male	ESP	Local	Coastal	Native	Joy	18-29	High Sch.	<14	2000s	5	3	4	4	5	4	4	5	5	5	4.4
AC182	Cadaqués	Male	ESP	Local	Coastal	Native	Joy	30-39	Master	14-35	1990s	4	3	3	4	3	4	4	4	3	4	3.6
AC183	Cadaqués	Male	ESP	Local	Coastal	Native	Joy	18-29	University	<14	2000s	3	3	2	3	3	4	3	3	3	4	3.1
AC184	Cadaqués	Female	ESP	Local	Coastal	Native	Strong	40-49	Master	14-35	1980s	2	4	2	3	4	5	4	4	3	4	3.5
ZC09	Cadaqués	Male	ESP	Local	Coastal	Non-native	Joy	40-49	University	<14	2000s	5	3	4	4	5	5	3	3	5	5	4.2
ZC17	Cadaqués	Female	ESP	Local	Coastal	Non-native	Strong	70-79	High Sch.	<14	1940s	5	5	5	1	2	5	1	1	2	2	2.9
ZC19	Cadaqués	Female	FRA	Tourist	Inland	Frequent	Strong	40-49	Master	<14	1990s	3	3	3	2	4	4	3	3	4	5	3.4
ZC22	Cadaqués	Male	ESP	Local	Coastal	Non-native	Strong	50-59	Middle Sch.		1960s	1	5	5	5	5	5	3	5	5	5	4.4
MC187	Cadaqués	Female	ESP	Local	Coastal	Native	Strong	30-39	University	14-35	1990s	4	5	3	3	5	5	5	3	5	5	4.3
MC180	Cadaqués	Female	ESP	Local	Coastal	Native	Strong	18-29	University	<14	2000s	4	5	4	4	5	4	4	4	3	4	4.1
MC181	Cadaqués	Male	ESP	Local	Coastal	Native	Strong	40-49	Prof./Tech.	14-35	1980s	4	5	4	4	5	5	4	4	4	3	4.2
MC182	Cadaqués	Female	ESP	Tourist	Inland	Frequent	Joy	18-29	Middle Sch.	No income	2000s	3	5	4	4	5	3	5	3	5	5	4.2
MC183	Cadaqués	Male	ESP	Local	Coastal	Native	Need	50-59	University	35-56	1970s	1	5	5	5	5	5	4	4	5	5	4.4
MC184	Cadaqués	Male	ESP	Local	Coastal	Native	Strong	60-69	University	14-35	1960s	2	5	1	5	5	5	5	4	5	5	4.2
MC185	Cadaqués	Female	ESP	Local	Coastal	Native	Joy	18-29	Middle Sch.	No income	2000s	4	3	2	1	5	5	4	4	4	4	3.6
MC186	Cadaqués	Male	ESP	Local	Coastal	Native	Strong	40-49	University	14-35	1980s	2	4	4	4	4	4	4	4	4	2	3.6
MC188	Cadaqués	Male	ESP	Local	Coastal	Native	Strong	18-29	University	<14	2000s	5	5	2	2	5	3	4	3	5	4	3.8
MC189	Cadaqués	Female	ESP	Tourist	Coastal	Frequent	Strong	50-59	Master	35-56	1960s	3	4	1	2	4	4	4	4	4	4	3.4
MC190	Cadaqués	Female	ESP	Tourist	Near coast	Frequent	Need	30-39	University	14-35	2000s	3	4	4	4	5	4	3	3	4	4	3.8
MC191	Cadaqués	Male	ESP	Tourist	Coastal	Frequent	Strong	40-49	Prof./Tech.	14-35	1990s	4	5	4	4	5	5	3	4	5	5	4.4
MC194	Cadaqués	Female	ESP	Tourist	Coastal	Frequent	Strong	50-59	University	35-56	1980s	4	5	4	4	4	5	5	2	4	4	4.1
ZP01	Port Selva	Male	ESP	Tourist	Coastal	Frequent	Joy	60-69	Master	35-56	2000s	1	3	2	1	5	4	3	4	4	4	3.1
ZP02	Port Selva	Male	ESP	Tourist	Coastal	Frequent	Strong	18-29	Primary Sch.	14-35	1990s	5	5	1	1	5	5	5	5	5	1	3.8
UR21	Roses	Male	ESP	Local	Coastal	Non-native	Joy	30-39	High Sch.	14-35	2000s	4	5	2	1	5	2	3	1	5	1	2.9
UR20	Roses	Male	FRA	Tourist	Inland	Frequent	Strong	60-69	Prof./Tech.	14-35	1990s	1	5	4	1	5	5	2	5	5	5	3.8
MC232	Cadaqués	Female	ESP	Local	Coastal	Non-native	Strong	50-59	University	<14	2000s	3	5	3	3	4	5	4	3	4	4	3.8
MC230	Cadaqués	Female	ESP	Local	Coastal	Non-native	Strong	40-49	Master	<14	2000s	5	5	4	5	5	5	3	3	5	5	4.5
AP201	Port Selva	Female	ESP	Tourist	Near coast	Frequent	Need	40-49	Master	14-35	2000s	3	4	4	4	5	3	4	4	3	4	3.8
AP202	Port Selva	Male	ESP	Tourist	Near coast	Frequent	Need	50-59	Prof./Tech.	14-35	2000s	2	5	4	3	4	2	4	5	4	3	3.6
AP203	Port Selva	Female	ESP	Tourist	Coastal	Frequent	Joy	30-39	Master		2000s	4	4	5	5	3	4	4	2	3	4	3.8
AP204	Port Selva	Male	ESP	Tourist	Coastal	Frequent	Joy	30-39	Master	35-56	2000s	3	4	4	4	5	4	3	3	4	4	3.8
AP206	Port Selva	Female	ESP	Tourist	Inland	Frequent	Joy	50-59	Middle Sch.		1970s	4	3	4	4	4	4	3	3	4	4	3.7
AP208	Port Selva	Male	ESP	Tourist	Coastal	Frequent	Need	18-29	University	No income	2000s	4	3	4	4	4	4	4	4	4	5	4.0
AP209	Port Selva	Female	FRA	Tourist	Inland	Frequent	Need	40-49	Master	35-56	1970s	4	4	4	5	5	5	5	5	5	5	4.7
AR186	Roses	Female	GBR	Tourist	Inland	Frequent	Joy	80-89	High Sch.	<14	1980s	1	4	4	3	1	3	5	4	3	4	3.2
AR190	Roses	Male	ESP	Tourist	Near coast	Infrequent	Joy	50-59	High Sch.	35-56	1960s	4	4	2	3	5	4	4	2	4	3	3.5
AR191	Roses	Male	ESP	Local	Coastal	Non-native	Strong	80-89	Primary Sch.		1960s	4	5	2	2	5	4	4	4	3	4	3.7
AR192	Roses	Male	BEL	Tourist	Inland	Frequent	Need	50-59	Middle Sch.	14-35	1990s	4	4	2	4	4	5	4	4	4	4	3.9
AR193	Roses	Female	FRA	Tourist		Frequent	Need	50-59	Master	35-56	1970s	3	5	3	2	4	4	4	3	3	4	3.5
AR198	Roses	Female	FRA	Tourist	Inland	Infrequent	Joy	40-49	University	35-56	2000s	4	5	4	3	5	5	4	3	3	5	4.1

				Sociodemographic parameters								Statements (See SM 2 for the entire statement)										
Code	Place of interview	Gender	Country	Local / Tourist	Residence-to-sea distance	Frequency & Antiquity	Marine affinity/ dependence	Age group	Education level	Income	Baseline decade	Hot Tº	Marine life	Respect towards nature	Clean coasts	Marine pollution	Boat transit & anchor	Invasive species	Overfish	Nostalgia	Tourism	Average
MR195	Roses	Female	ESP	Local	Coastal	Native	Strong	30-39	High Sch.	<14	1990s	4	5	3	2	4	4	3	3	4	2	3.4
MR196	Roses	Female	ESP	Local	Coastal	Non-native	Strong	50-59	High Sch.	14-35	1980s	2	3	2	4	4	4	4	3	3	4	3.3
MR197	Roses	Female	ESP	Tourist	Coastal	Frequent	Strong	30-39	Master	35-56	2000s	1	5	4	3	5	5	3	4	3	3	3.6
MR198	Roses	Female	ESP	Local	Coastal	Native	Strong	18-29	Master	14-35	2000s	4	5	3	3	3	4	4	3	3	5	3.7
MR200	Roses	Female	ESP	Local	Coastal	Native	Strong	50-59	High Sch.	14-35	1970s	4	5	4	2	5	5	5	3	5	5	4.3
MR201	Roses	Male	FRA	Tourist	Inland	Frequent	Joy	50-59	Master	35-56	1990s	4	1	2	3	4	3	5	4	2	4	3.2
MR202	Roses	Female	ESP	Local	Coastal	Native	Strong	18-29	Middle Sch.	No income	2000s	3	2	2	2	1	3	5	5	3	3	2.9
MR205	Roses	Male	ESP	Local	Coastal	Native	Joy	30-39	Prof./Tech.	<14	1990s	4	4	1	4	4	5	4	4	4	4	3.8
AC172	Cadaqués	Male	FRA	Tourist	Inland	Infrequent	Need	40-49	University	14-35	1990s	4	5	4	2	4	4	3	3	4	4	3.7
AC176	Cadaqués	Male	ESP	Local	Coastal	Native	Strong	40-49	Master	>56	1980s	3	4	4	1	5	2	5	3	2	1	3.0
AC177	Cadaqués	Female	ESP	Local	Coastal	Native	Strong	40-49	High Sch.	14-35	1980s	3	4	2	3	4	4	4	3	3	3	3.3
AC179	Cadaqués	Female	ESP	Tourist	Coastal	Frequent	Need	18-29	Master	<14	1990s	5	5	2	2	5	5	4	5	3	5	4.1
AC181	Cadaqués	Female	ESP	Local	Coastal	Native	Joy	50-59	Prof./Tech.	14-35	1970s	4	3	3	4	3	4	4	4	3	4	3.6
MP243	Port Selva	Male	FRA	Tourist	Inland		Joy	40-49	University	14-35		5	5	2	3	3	4	3	3	2	3	3.3
MP244	Port Selva	Male	ESP	Tourist	Near coast	Frequent	Strong	60-69	University	35-56	1970s	4	5	2	2	5	3	3	5	3	2	3.4
MP245	Port Selva	Male	ESP	Local	Coastal	Native	Strong	30-39	Middle Sch.	14-35	1990s	4	5	2	5	5	5	5	5	4	4	4.4
MP246	Port Selva	Female	ESP	Local	Coastal	Native	Strong	50-59	Prof./Tech.	14-35	1970s	4	5	2	2	5	4	4	2	3	4	3.5
MP248	Port Selva	Male	FRA	Tourist	Inland	Frequent	Joy	40-49	Master	>56	2000s	1	3	2	2	3	4	2	4	2	4	2.7
MP249	Port Selva	Male	ESP	Tourist	Coastal	Infrequent	Joy	18-29	High Sch.	<14	1990s	4	3	1	2	5	5	4	3	3	3	3.3
MP150	Port Selva	Male	ESP	Tourist	Coastal	Frequent	Strong	60-69	Master	35-56	1990s	2	3	2	2	2	4	4	4	3	3	2.9
MP252	Port Selva	Female	CHE	Tourist	Inland	Frequent	Need	18-29	High Sch.	No income	2000s	3	5	2	3	4	3	5	4	4	5	3.8
MP256	Port Selva	Female	ESP	Tourist	Near coast	Frequent	Need	18-29	High Sch.	No income	2000s	4	5	3	2	5	4	3	3	3	5	3.7
MP255	Port Selva	Female	ESP	Tourist	Coastal	Frequent	Strong	18-29	High Sch.	No income	2000s	5	5	4	4	3	4	3	4	4	5	4.1
UC12B	Cadaqués	Male	ESP	Local	Coastal	Non-native	Strong	60-69	PhD	>56	1970s	5	5	1	3	5	5	4	2	4	5	3.9
UC14	Cadaqués	Female	ESP	Tourist	Coastal	Frequent	Strong	18-29	University	No income	1990s	3	5	2	2	5	4	3	2	5	5	3.6
UC16	Cadaqués	Male	ESP	Local	Coastal	Non-native	Need	50-59	University	No income	1990s	1	5	2	5	5	5	3	1	1	5	3.3
UC16B	Cadaqués	Male	ESP	Tourist	Coastal	Frequent	Need	40-49	University	>56	2000s	2	3	3	2	2	4	3	2	2	4	2.7
UC11B	Cadaqués	Male	ESP	Local	Coastal	Non-native	Strong	18-29	Middle Sch.	14-35	2000s	3	5	5	4	1	5	5	3	4	4	3.9
UC11	Cadaqués	Female	FRA	Tourist	Coastal	Infrequent	Strong	30-39	Master	14-35	2000s	2	4	2	2	3	4	4	4	4	4	3.3
MC220	Cadaqués	Male	ESP	Local	Coastal	Non-native	Need	50-59	Middle Sch.	No income	1980s	2	5	5	5	4	5	4	4	5	2	4.1
MC221	Cadaqués	Male	ESP	Local	Coastal	Native	Joy	30-39	High Sch.	14-35	1990s	4	5	2	3	5	5	5	3	3	4	3.9
MP212	Port Selva	Female	ESP	Tourist	Coastal	Frequent	Strong	18-29	Master	No income	1990s	4	5	4	3	5	4	3	5	5	4	4.2
MP210	Port Selva	Male	ESP	Local	Coastal	Native	Strong	60-69	High Sch.		1960s	5	5	5	5	5	5	5	5	5	5	5.0
MC223	Cadaqués	Male	ITA	Tourist	Inland	Infrequent	Need	30-39	High Sch.	14-35	2000s	3	3	3	1	3	2	4	3	3	3	2.8
UC01	Cadaqués	Female	ESP	Tourist	Near coast	Infrequent	Joy	50-59	High Sch.	<14	1980s	5	5	5	1	4	5	5	5	5	5	4.5
UC02	Cadaqués	Female	ESP	Tourist	Coastal	Frequent	Joy	70-79	Prof./Tech.		1980s	3	5	1	2	5	3	2	1	4	3	2.9
UC04	Cadaqués	Male	FRA	Tourist	Inland	Frequent	Joy	60-69	University	14-35	1980s	4	5	1	1	4	3	3	3	5	5	3.4
UC07	Cadaqués	Female	FRA	Tourist	Inland	Infrequent	Joy	50-59	Master	14-35	2000s	4	3	4	2	4	4	4	3	3	4	3.5
MC105	Cadaqués	Female	ESP	Local	Coastal	Native		40-49	University	14-35	1980s	3	5	5	3	4	4	3	4	5	3	3.9
MR240	Roses	Male	ESP	Local	Coastal	Native	Joy	60-69	High Sch.	14-35	1960s	3	4	2	1	2	3	4	3	2	2	2.6
MR241	Roses	Female	ESP	Local	Coastal	Non-native	Strong	18-29	Prof./Tech.	14-35	1990s	2	4	3	3	4	4	4	3	4	4	3.5
UR31	Roses	Female	FRA	Tourist	Inland	Frequent	Need	70-79	Middle Sch.	35-56	1970s	3	4	2	2	4	4	3	2	3	4	3.1
UR27	Roses	Male	FRA	Tourist	Inland	Frequent	Strong	50-59	High Sch.	>56	2000s	1	4	2	2	4	2	4	5	3	3	3.0
UR26	Roses	Male	ESP	Local	Coastal	Native	Joy	60-69	High Sch.	14-35	1960s	4	3	1	1	2	2	3	2	3	3	2.4
UR23	Roses	Male	ESP	Local	Coastal	Non-native	Strong	40-49	University	14-35	1990s	2	4	3	2	4	5	5	5	4	5	3.9
WP999	Port Selva	Female	ITA	Tourist	Near coast	Frequent	Joy	70-79	University	>56	1950s	2	4	2	2	4	4	4	4	2	2	3.0
MC16	Cadaqués	Male	ESP	Local	Coastal	Native	Joy	40-49	University	14-35	1980s	1	3	3	2	3	1	3	1	1	1	1.9
MC20	Cadaqués	Male	ESP	Local	Coastal	Native	Joy	18-29	Middle Sch.	<14	2000s	1	2	5	2	3	4	2	3	2	3	2.7
MC22	Cadaqués	Female	ESP	Local	Coastal	Native	Strong	18-29	University	<14	2000s	3	5	4	2	5	4	3	3	4	4	3.7
MC18	Cadaqués	Male	ESP	Local	Coastal	Native	Strong	40-49	Master	14-35	1980s	3	5	2	3	4	5	4	5	4	5	4.0
MP257	Port Selva	Female	ESP	Local	Coastal	Native	Joy	50-59	Primary Sch.		1970s	4	5	4	4	4	5	3	5	4	3	4.1
MP258	Port Selva	Female	ESP	Tourist	Near coast	Frequent	Need	50-59	Prof./Tech.	<14	1960s	3	5	3	3	4	5	4	5	4	5	4.1
MP259	Port Selva	Female	ESP	Tourist	Coastal	Frequent	Joy	40-49	University	14-35	1990s	4	4	2	2	4	4	4	4	3	4	3.5
UP41	Port Selva	Male	FRA	Tourist	Coastal	Frequent	Joy	30-39	High Sch.	14-35	1980s	2	5	2	1	5	1	3	3	1	3	2.6
UP46	Port Selva	Female	ESP	Tourist	Near coast	Frequent	Strong	40-49	University	>56	1990s	5	4	2	1	4	5	5	5	4	5	4.0
UP50	Port Selva	Female	ESP	Local	Coastal	Native	Strong	50-59	High Sch.		1970s	4	4	2	4	4	5	3	1	2	3	3.2
UP52	Port Selva	Male	ESP	Tourist	Near coast	Frequent	Need	60-69	Master	>56	1990s	4	5	1	2	1	4	5	4	4	5	3.5
UP53	Port Selva	Female	ESP	Tourist	Near coast	Frequent	Joy	60-69	University	14-35	1990s	5	5	1	1	4	4	5	3	4	3	3.5
UP56	Port Selva	Male	ESP	Local	Coastal	Native	Strong	40-49	Prof./Tech.	14-35	1980s	5	5	1	1	5	5	5	3	5	4	3.9
UP58	Port Selva	Male	ESP	Local	Coastal	Non-native	Need	40-49	University	35-56	1990s	3	5	2	3	5	3	3	5	1	5	3.5
UP59	Port Selva	Male	BEL	Tourist	Inland	Frequent	Strong	40-49	Middle Sch.		1990s	1	5	4	3	5	1	4	5	4	3	3.5
ZP03	Port Selva	Female	USA	Tourist	Coastal	Frequent	Joy	70-79	Master	>56	1980s	2	5	3	5	5	5	5	5	5	5	4.5
ZP04	Port Selva	Female	ESP	Local	Coastal	Native	Need	40-49	Master	14-35	1980s	2	5	2	1	5	3	4	4	2	2	3.0
ZP05	Port Selva	Female	ESP	Tourist	Near coast	Frequent	Need	30-39	Prof./Tech.		1980s	2	4	4	4	4	4	4	4	4	4	3.8
ZP06	Port Selva	Male	ESP	Tourist	Coastal	Frequent	Strong	80-89	High Sch.		1950s	4	4	2	2	4	4	4	4	4	4	3.6
ZP07	Port Selva	Male	ESP	Tourist	Coastal	Frequent	Joy	40-49	Master	>56	1980s	4	5	4	4	5	5	3	3	4	2	3.9
ZP08	Port Selva	Male	ESP	Local	Coastal	Non-native	Strong	40-49	High Sch.	14-35	1970s	2	5	3	1	1	5	5	5	3	3	3.3
MP170	Port Selva	Male	ESP	Tourist	Coastal	Frequent	Strong	70-79	Middle Sch.		1990s	3	5	3	3	5	5	3	5	5	4	4.1
MP171	Port Selva	Male	ESP	Local	Coastal	Non-native	Strong	18-29	Middle Sch.	<14	2000s	3	4	4	4	5	3	1	4	3	5	3.6
MP174	Port Selva	Female	ESP	Tourist	Coastal	Frequent	Joy	18-29	University	<14	1990s	3	5	3	2	5	4	3	3	4	3	3.5
MP175	Port Selva	Female	ESP	Local	Coastal	Native	Strong	40-49	University	<14	1980s	3	5	2	1	4	4	3	4	3	3	3.2
MP176	Port Selva	Female	ESP	Tourist	Coastal	Frequent	Joy	18-29	University	14-35	2000s	4	5	2	2	4	4	3	4	4	4	3.6
MP177	Port Selva	Male	ESP	Local	Coastal	Native	Strong	18-29	University	14-35	2000s	5	5	4	4	5	5	2	2	1	4	3.7
AP164	Port Selva	Male	ESP	Tourist	Near coast	Frequent	Joy	70-79	Prof./Tech.	14-35	1940s	4	3	2	2	4	4	2	5	4	3	3.3
AP165	Port Selva	Female	ESP	Tourist	Near coast	Frequent	Strong	70-79	University		1960s	2	4	2	2	4	5	3	4	3	4	3.3
AP170	Port Selva	Female	ESP	Local	Coastal	Native	Strong	40-49	High Sch.	<14	1980s	2	5	4	3	5	4	2	4	3	3	3.5
AR144	Roses	Female	ESP	Local	Coastal	Native	Joy	18-29	Prof./Tech.	<14	2000s	4	3	2	4	5	3	3	2	3	4	3.3
AR145	Roses	Female	ESP	Local	Coastal	Native	Joy	30-39	Prof./Tech.	14-35	1990s	1	3	3	2	3	3	3	5	3	2	2.8
AR146	Roses	Female	ESP	Local	Coastal	Native	Need	30-39	Prof./Tech.	<14	1990s	3	5	3	3	3	5	4	4	5	4	3.9
AR147	Roses	Female	ESP	Local	Coastal	Native	Joy	40-49	Middle Sch.	14-35	1980s	1	3	2	2	5	4	4	1	3	3	2.8
AR149	Roses	Female	FRA	Tourist	Inland		Joy	70-79	Middle Sch.	<14		3	4	2	2	4	4	4	4	4	4	3.5
AR153	Roses	Male	ESP	Local	Coastal	Native	Need	18-29	Primary Sch.	14-35	2000s	4	4	3	2	5	4	3	4	4	4	3.7
SC17	Cadaqués	Male	FRA	Tourist	Near coast		Joy	70-79	Middle Sch.	14-35		4	4	3	2	3	5	4	3	4	3	3.5
SC18	Cadaqués	Female	ESP	Tourist	Near coast	Frequent	Joy	40-49	University	<14	1990s	4	4	4	3	4	4	3	4	4	4	3.8
SC19	Cadaqués	Male	ESP	Tourist	Near coast	Frequent	Need	40-49	University	14-35	1990s	5	4	5	2	5	5	5	4	3	4	4.2
SC20	Cadaqués	Female	FRA	Tourist	Inland	Frequent	Need	50-59	High Sch.	35-56	1990s	3	4	4	2	3	4	4	3	4	4	3.5
SC21	Cadaqués	Male	MAR	Tourist	Coastal		Strong	50-59	Primary Sch.	<14		5	5	2	2	5	1	5	5	5	4	3.9
SC22	Cadaqués	Female	FRA	Tourist	Inland	Frequent	Need	50-59	Prof./Tech.		1990s	2	4	3	4	4	4	4	4	4	4	3.7
SC23	Cadaqués	Female	GBR	Tourist	Coastal		Joy	40-49	University	>56		4	4	1	4	1	5	3	4	4	4	3.4
SC25	Cadaqués	Female	ESP	Local	Coastal	Native	Indifferent	70-79	Primary Sch.	14-35	1950s	5	5	2	1	5	5	5	1	4	2	3.5
SC26	Cadaqués	Male	ESP	Local	Coastal	Native	Joy	80-89	Primary Sch.	14-35	1940s	5	5	2	1	4	4	5	3	3	2	3.4
MC02	Cadaqués	Female	FRA	Tourist			Joy	40-49	Master	14-35		4	4	2	2	4	2	3	3	2	2	2.8
MC05	Cadaqués	Female	ESP	Local	Coastal	Native	Indifferent	18-29	High Sch.	No income	2000s	4	5	4	3	5	4	3	4	5	5	4.2
MC06	Cadaqués	Female	ESP	Tourist	Coastal	Frequent	Joy	50-59	University	35-56	1970s	4	4	4	4	3	4	4	5	3	5	4.0
MC08	Cadaqués	Female	ESP	Local	Coastal	Native	Joy	40-49	University		1980s	3	5	3	3	4	5	4	5	5	4	4.1
MC07	Cadaqués	Male	ESP	Tourist	Coastal	Frequent	Joy	50-59	University	>56	1980s	4	4	4	4	4	4	3	3	4	5	3.9
MC09	Cadaqués	Male	ESP	Local	Coastal	Native	Joy	60-69	High Sch.	<14	1960s	5	5	4	4	4	5	4	5	3	5	4.4
MC10	Cadaqués	Female	ESP	Local	Coastal	Native	Joy	60-69	University	<14	1960s	5	5	3	4	4	5	3	3	5	4	4.1
MC11	Cadaqués	Female	ARG	Tourist	Coastal	Infrequent	Strong	30-39	University	<14	2000s	3	4	2	3	4	5	3	3	4	5	3.6
MC15	Cadaqués	Male	ESP	Local	Coastal	Native	Need	18-29	Prof./Tech.	14-35	2000s	2	5	4	3	5	5	3	3	4	3	3.7
AR158	Roses	Female	ESP	Local	Coastal	Native	Strong	18-29	High Sch.	<14	2000s	3	4	4	5	5	3	3	3	5	5	4.0
AR162	Roses	Female	FRA	Tourist	Coastal	Frequent	Joy	70-79	No Sch.		1970s	5	5	2	2	3	5	4	5	5	5	4.1
AP125	Port Selva	Male	ESP	Local	Coastal	Native	Strong	70-79	PhD	14-35	1950s	4	5	2	2	4	4	3	4	4	4	3.6
AP126	Port Selva	Female	FRA	Tourist	Near coast	Frequent	Indifferent	60-69	No Sch.		1990s	4	4	2	2	5	1	4	4	4	3	3.3
AP127	Port Selva	Female	ESP	Tourist	Near coast	Frequent	Joy	60-69	University		1980s	4	3	2	2	4	2	3	2	2	2	2.6
AP128	Port Selva	Female	ESP	Local	Coastal		Indifferent	18-29	High Sch.	<14		4	5	3	2	5	4	2	3	1	2	3.1
AP129	Port Selva	Male	ESP	Tourist	Coastal	Frequent	Joy	18-29	High Sch.	14-35	2000s	3	5	4	5	3	5	3	5	4	5	4.2

				**Sociodemographic parameters**								**Statements** (See SM 2 for the entire statement)										
**Code**	**Place of interview**	**Gender**	**Country**	**Local / Tourist**	**Residence-to-sea distance**	**Frequency & Antiquity**	**Marine affinity/ dependence**	**Age group**	**Education level**	**Income**	**Baseline decade**	**Hot T°**	**Marine life**	**Respect towards nature**	**Clean coasts**	**Marine pollution**	**Boat transit & anchor**	**Invasive species**	**Overfish**	**Nostalgia**	**Tourism**	**Average**
AP131	Port Selva	Female	ESP	Tourist	Near coast	Frequent	Joy	60-69	Master		2000s	4	3	2	2	3	1	3	3	1	1	2.3
AP135	Port Selva	Male	ESP	Tourist	Near coast	Frequent	Need	50-59	High Sch.	35-56	1990s	4	4	4	3	4	5	4	3	3	5	3.9
AP139	Port Selva	Male	ESP	Local	Coastal	Native	Joy		Prof./Tech.	14-35		1	2	5	4	5	5	5	5	3	4	3.9
AP140	Port Selva	Female	ESP	Tourist	Inland	Frequent	Strong	40-49	University	14-35	2000s	4	5	3	3	5	5	5	4	5	5	4.4
AR108	Roses	Male	ESP	Local	Coastal	Native	Indifferent	60-69	University	35-56	1960s	2	5	5	2	4	4	3	3	4	4	3.6
WP103	Port Selva	Male	ESP	Tourist	Near coast	Frequent	Joy	40-49	Prof./Tech.	14-35	1980s	1	2	4	4	4	4	3	2	2	3	2.9
WP104	Port Selva	Female	ESP	Tourist	Coastal	Frequent	Strong		Master	14-35	2000s	4	5	5	5	1	5	5	5	5	5	4.5
WP105	Port Selva	Female	FRA	Tourist	Inland	Infrequent	Indifferent	30-39	University	>56	2000s	3	5	2	3	3	3	3	4	5	4	3.5
WP106	Port Selva	Male	GBR	Tourist	Inland	Infrequent	Joy	40-49	Master	>56	2000s	5	5	3	3	4	5	5	5	4	4	4.3
WP112	Port Selva	Female	ESP	Local	Near coast	Non-native	Joy	50-59	High Sch.		1960s	3	5	4	3	5	4	4	1	3	4	3.6
WP113	Port Selva	Male	ESP	Tourist	Near coast	Frequent	Joy	60-69	PhD	14-35	1970s	3	5	1	3	4	5	4	4	3	4	3.6
WP116	Port Selva	Male	ESP	Tourist	Coastal	Infrequent	Need	18-29	University	<14	2000s	4	5	1	2	4	5	3	3	5	5	3.7
MR162	Roses	Male	ESP	Local	Coastal	Non-native	Strong	40-49	Master	35-56	1990s	4	5	3	2	5	4	4	4	4	5	4.0
MR163	Roses	Male	ESP	Local	Coastal	Native	Strong	30-39	Prof./Tech.	14-35	1990s	1	5	4	5	5	5	3	2	5	3	3.8
MR165	Roses	Male	BEL	Tourist	Coastal	Frequent	Joy	40-49	Prof./Tech.		1980s	3	2	2	3	3	4	3	3	3	3	2.9
MR164	Roses	Male	BEL	Tourist	Coastal	Frequent	Joy	30-39	Middle Sch.	>56	2000s	2	2	2	3	3	4	3	3	3	3	2.8
MR165	Roses	Female	ESP	Tourist	Inland	Frequent	Joy	50-59	Primary Sch.	14-35	1980s	5	5	4	5	5	1	1	2	1	1	3.0
MR166	Roses	Male	ESP	Tourist	Inland	Frequent	Joy	50-59	Primary Sch.	14-35	1980s	5	5	5	4	5	5	5	4	5	4	4.7
MP130	Port Selva	Male	ESP	Local	Coastal	Native	Strong	70-79	Middle Sch.	35-56	1950s	4	5	4	2	5	5	3	4	5	4	4.1
MP131	Port Selva	Female	ESP	Local	Coastal	Native	Strong	40-49	University	35-56	1980s	1	4	2	2	5	4	3	4	4	4	3.3
MP132	Port Selva	Female	ESP	Local	Coastal	Non-native	Strong	50-59	Prof./Tech.	14-35	1990s	5	5	5	2	5	5	5	3	5	5	4.5
MP134	Port Selva	Male	ESP	Local	Coastal	Native	Strong	18-29	Master	<14	2000s	4	5	4	5	5	3	3	3	4	3	3.9
MP140	Port Selva	Female	ESP	Local	Coastal	Native	Strong	50-59	Prof./Tech.	<14	1970s	4	4	3	2	3	4	4	4	4	3	3.5
MP141	Port Selva	Female	ESP	Tourist	Coastal	Frequent	Joy	30-39	University		1980s	4	3	5	5	5	5	3	3	1	4	3.8
MP142	Port Selva	Male	ESP	Local	Coastal	Native	Indifferent	50-59	University	14-35	1970s	5	3	4	3	5	4	4	4	4	4	4.0
MP143	Port Selva	Female	ESP	Local	Coastal	Native	Joy	18-29	University	14-35	2000s	5	5	4	3	5	4	4	4	3	5	4.2
MP144	Port Selva	Female	ESP	Tourist	Coastal		Strong	30-39	Prof./Tech.	<14		5	5	5	3	3	5	3	5	5	4	4.3
ZC24	Cadaqués	Female	ESP	Local	Coastal	Non-native	Need	18-29	High Sch.	No income	2000s	3	5	5	4	4	5	3	3	5	5	4.2
ZC25	Cadaqués	Male	ESP	Tourist	Coastal	Frequent	Joy	18-29	Master	<14	1990s	2	5	2	5	5	5	3	4	4	5	4.0
ZC26	Cadaqués	Male	ESP	Local	Coastal	Non-native	Strong	18-29	Prof./Tech.	<14	2000s	4	5	5	3	5	5	5	5	5	5	4.7
ZC27	Cadaqués	Female	ESP	Local	Coastal	Non-native	Joy	50-59	Master	35-56	1980s	4	4	2	2	3	4	3	3	3	4	3.2
ZC33	Cadaqués	Male	ESP	Tourist	Coastal	Frequent	Need	70-79	High Sch.	>56	1990s	5	5	5	2	3	5	3	4	4	5	4.1
UC68	Cadaqués	Female	ESP	Tourist	Near coast	Frequent	Need	40-49	University	14-35	1990s	3	5	2	4	5	5	3	5	5	5	4.2
UC69	Cadaqués	Female	ESP	Tourist	Coastal	Frequent	Strong	30-39	Prof./Tech.	14-35	1990s	4	5	4	3	5	5	3	2	4	5	4.0
MC260	Cadaqués	Female	ESP	Tourist	Coastal	Frequent	Joy	18-29	High Sch.	No income	1990s	4	3	4	4	4	5	4	5	2	5	4.0
MC261	Cadaqués	Female	ESP	Local	Coastal	Native	Strong	30-39	Master	14-35	1990s	4	5	3	5	5	5	5	5	5	5	4.7
MC262	Cadaqués	Male	ESP	Local	Coastal	Native	Strong	30-39	Prof./Tech.	14-35	1990s	3	5	5	4	5	5	4	3	5	5	4.4
MC263	Cadaqués	Male	ESP	Local	Coastal	Native	Strong	30-39	High Sch.	<14	1990s	3	5	2	4	5	5	4	5	5	5	4.3
MC264	Cadaqués	Male	ESP	Local	Coastal	Native	Need	30-39	High Sch.	35-56	1990s	3	4	3	2	4	4	4	3	4	5	3.6
MC265	Cadaqués	Female	ESP	Local	Coastal	Native	Strong	40-49	Middle Sch.	<14	1980s	3	5	4	2	5	5	5	4	5	3	4.1
MC266	Cadaqués	Female	ESP	Local	Coastal	Native	Strong	18-29	Middle Sch.	No income	2000s	5	4	3	5	5	5	5	5	3	3	4.3
MC267	Cadaqués	Male	ESP	Local	Coastal	Native	Joy	18-29	Middle Sch.	14-35	2000s	3	5	5	5	5	5	3	5	5	5	4.6
MC268	Cadaqués	Female	ESP	Tourist	Coastal	Frequent	Strong	18-29	University	<14	2000s	5	5	4	4	4	4	4	4	5	5	4.4
WC75	Cadaqués	Female	ESP	Tourist	Coastal	Frequent	Joy	50-59	University	14-35	1970s	2	3	4	4	4	4	3	2	3	4	3.3
WC76	Cadaqués	Female	ESP	Tourist	Near coast	Frequent	Need	70-79	High Sch.		1940s	3	5	2	4	4	4	4	3	3	4	3.6
WC77	Cadaqués	Female	FRA	Tourist	Inland	Infrequent	Need	40-49	University	35-56	2000s	3	4	1	2	3	3	3	4	4	3	3.0
WC78	Cadaqués	Male	FRA	Tourist	Inland	Infrequent	Joy	40-49	Prof./Tech.	14-35	2000s	3	4	2	3	3	4	3	4	4	4	3.4
WC79	Cadaqués	Male	ESP	Tourist	Near coast	Frequent	Joy	60-69	High Sch.	14-35	2000s	4	5	2	5	5	5	3	2	4	4	3.9
MC100	Cadaqués	Male	ESP	Local	Coastal	Native	Avoid	40-49	Primary Sch.		1980s	3	5	5	5	5	5	3	2	5	5	4.3
MC101	Cadaqués	Female	ESP	Local	Coastal	Native	Need	18-29	University	<14	2000s	4	5	5	4	5	5	3	3	4	5	4.3
MC104	Cadaqués	Female	ESP	Tourist	Coastal	Frequent	Need	40-49	High Sch.	14-35	1980s	4	5	3	2	4	5	5	3	3	3	3.7
MC106	Cadaqués	Male	ESP	Tourist	Coastal	Frequent	Strong	18-29	University		1990s	5	3	2	4	3	4	5	4	5	5	4.0
MC107	Cadaqués	Female	ESP	Local	Coastal	Non-native	Need	40-49	Prof./Tech.	<14	1990s	3	4	2	3	4	4	3	3	4	5	3.5
MC110	Cadaqués	Female	ESP	Local	Coastal	Native	Strong	50-59	University	35-56	1970s	1	3	2	4	5	1	1	5	2	3	2.7
MC111	Cadaqués	Female	ESP	Local	Coastal	Native	Strong	18-29	Prof./Tech.	<14	2000s	2	4	3	5	5	5	3	3	5	5	4.0
WP54	Port Selva	Female	GBR	Tourist	Near coast	Frequent	Joy	30-39	PhD		2000s	3	5	2	2	3	4	3	3	4	5	3.4
WP55	Port Selva	Male	ESP	Tourist	Coastal	Frequent	Need	40-49	University	<14	1970s	3	5	2	4	3	4	4	4	4	4	3.7
WP56	Port Selva	Female	ESP	Tourist	Near coast	Infrequent	Need	50-59	Master		1990s	1	4	3	3	4	5	3	5	4	5	3.7
WP57	Port Selva	Male	ESP	Tourist	Coastal	Frequent	Need	40-49	University	35-56	1980s	4	5	4	3	2	5	3	4	5	5	4.0
WP58	Port Selva	Female	ESP	Tourist	Coastal	Frequent	Need	50-59	University	35-56	1990s	4	5	3	3	4	5	4	3	4	5	4.0
WP60	Port Selva	Female	ESP	Tourist	Inland	Infrequent	Need	18-29	University	<14	1990s	5	5	4	5	5	5	5	3	3	4	4.4
WP61	Port Selva	Female	ESP	Tourist	Inland	Frequent	Need	18-29	University	<14	2000s	5	4	4	5	4	5	5	4	5	5	4.6
WP66	Port Selva	Female	DEU	Tourist	Inland	Infrequent	Joy	50-59	University	<14	1990s	3	3	4	4	3	4	3	4	3	3	3.4
WP68	Port Selva	Female	FRA	Tourist	Coastal	Frequent	Need	40-49	Master		1980s	1	5	2	2	4	5	5	4	1	4	3.3
WP69	Port Selva	Male	FRA	Tourist	Inland	Frequent	Joy	40-49	Prof./Tech.	35-56	1980s	3	4	2	3	4	5	4	4	4	4	3.7
WP70	Port Selva	Female	ESP	Tourist	Near coast	Infrequent	Joy	50-59	High Sch.		1980s	4	3	2	2	2	4	3	3	2	2	2.7
MP90	Port Selva	Male	ESP	Local	Coastal	Native	Need	70-79	Middle Sch.	<14	1950s	3	5	2	1	5	2	3	4	4	5	3.4
MP91	Port Selva	Male	ESP	Local	Coastal	Non-native	Strong	60-69	Prof./Tech.		1970s	5	5	5	5	5	4	4	2	3	1	3.9
MP92	Port Selva	Female	ESP	Local	Coastal	Native	Strong	40-49	Prof./Tech.		1980s	4	5	5	4	5	5	4	3	5	4	4.4
MP93	Port Selva	Male	FRA	Tourist	Inland		Avoid	50-59	University	<14		3	5	2	2	5	4	4	4	2	2	3.3
MP94	Port Selva	Female	ESP	Tourist	Coastal	Frequent	Strong	40-49	Prof./Tech.	14-35	2000s	4	3	2	2	5	5	3	4	3	3	3.4
AP75	Port Selva	Male	ESP	Tourist	Inland	Infrequent	Joy	40-49	Prof./Tech.	14-35	2000s	3	4	4	4	5	4	3	4	3	4	3.8
AP76	Port Selva	Male	FRA	Tourist	Inland	Frequent	Indifferent	40-49	PhD	35-56	1980s	3	2	2	1	2	5	3	3	3	3	2.7
AP77	Port Selva	Male	ESP	Tourist	Near coast	Frequent	Avoid	40-49	Prof./Tech.	14-35	2000s	4	5	2	3	3	5	5	4	5	5	4.1
AP78	Port Selva	Male	ESP	Local	Coastal	Native	Avoid	70-79	Primary Sch.		1950s	1	5	1	1	5	3	3	3	2	3	2.7
AP79	Port Selva	Female	ESP	Tourist	Near coast	Frequent		60-69			1930s	5	5	3	2	5	4	3	5	5	5	4.2
AAP80	Port Selva	Male	ESP	Local	Coastal	Non-native	Indifferent	50-59	Prof./Tech.	No income	1970s	5	5	5	5	5	5	5	5	3	5	4.8
AP81	Port Selva	Male	ESP	Tourist	Near coast	Frequent	Need	80-89	Middle Sch.	35-56	1950s	4	4	5	5	5	4	3	4	5	4	4.3
AP85	Port Selva	Female	ESP	Local	Coastal	Native	Joy	18-29	Prof./Tech.	<14	2000s	5	4	3	2	4	4	3	5	5	4	3.9
AP88	Port Selva	Female	ESP	Local	Coastal	Native	Strong	18-29	University	14-35	2000s	3	5	2	4	4	4	3	4	5	5	3.9
AP91	Port Selva	Male	ESP	Tourist	Near coast	Frequent	Joy	70-79	University	35-56	1960s	5	5	2	3	5	4	4	3	4	5	4.0
AP93	Port Selva	Female	ESP	Tourist	Coastal		Strong	18-29	University	No income		5	5	5	5	3	5	4	3	5	4	4.4
AP95	Port Selva	Female	ESP	Tourist	Coastal	Infrequent	Need	50-59	Master	>56		2	4	2	4	4	2	4	4	4	5	3.5
MR71	Roses	Female	ESP	Local	Coastal	Native	Avoid	18-29	University	<14	2000s	5	5	2	4	4	4	5	4	5	4	4.2
MR72	Roses	Female	ESP	Local	Coastal	Native	Strong	50-59	Middle Sch.	14-35	1970s	4	5	3	2	4	5	5	4	3	4	3.9
MR73	Roses	Male	ESP	Local	Coastal	Native	Strong	30-39	Middle Sch.	14-35	1990s	2	5	3	1	5	5	3	4	1	4	3.3
WR91	Roses	Male	FRA	Tourist	Inland	Infrequent	Joy	18-29	Master	14-35	2000s	4	4	4	4	5	4	3	3	4	5	4.0
WR93	Roses	Female	ESP	Local	Coastal	Non-native	Joy	50-59	University	14-35	2000s	2	4	1	1	2	5	3	4	3	3	2.8
WR95	Roses	Female	ESP	Local	Coastal	Native	Strong	60-69	University		1960s	5	5	4	3	5	5	3	1	3	4	3.8
WR96	Roses	Female	ESP	Local	Coastal	Native	Need	40-49	University	14-35	1980s	3	5	4	4	4	5	4	4	4	4	4.1
WR97	Roses	Female	ESP	Local	Near coast	Native	Joy	30-39	University		1990s	3	3	3	3	4	4	3	3	4	4	3.4
WR98	Roses	Female	ESP	Local	Coastal	Native	Joy	18-29	High Sch.	<14	2000s	4	5	5	5	5	4	3	3	5	5	4.4
WR99	Roses	Female	ESP	Local	Coastal	Native	Joy	30-39	Primary Sch.	<14	1990s	1	4	5	3	3	1	4	4	4	3	3.2
MR115	Roses	Female	ESP	Local	Coastal	Native	Strong	50-59	High Sch.	>56	1970s	3	5	4	1	5	4	2	4	4	5	3.7
MR116	Roses	Female	ESP	Local	Coastal	Native	Need	50-59	Prof./Tech.	14-35	1970s	5	5	2	3	5	5	3	5	5	5	4.3
MR117	Roses	Male	ESP	Local	Coastal	Native	Strong	30-39	Master	35-56	1990s	5	5	1	5	5	3	3	4	3	5	3.9
MR118	Roses	Female	ESP	Local	Coastal	Native	Strong	18-29	University	14-35	2000s	4	4	4	3	4	5	5	3	3	5	4.0
MR119	Roses	Female	ESP	Local	Coastal	Native	Need	18-29	University	<14	2000s	4	4	4	3	4	5	5	3	3	5	4.0
MR120	Roses	Male	BEL	Tourist	Inland	Frequent	Joy	40-49	Middle Sch.	35-56	1990s	4	3	2	2	2	3	4	4	3	3	3.0
MR121	Roses	Male	ESP	Local	Coastal	Native	Strong	50-59	Middle Sch.	14-35	1970s	5	5	2	1	5	5	5	4	5	5	4.2
MR125	Roses	Female	ESP	Local	Coastal	Native	Joy	18-29	Prof./Tech.	<14	2000s	4	5	1	4	5	5	3	4	4	4	3.9
MR124	Roses	Female	ESP	Local	Coastal	Native	Strong	18-29	Middle Sch.	<14	2000s	5	5	3	2	4	4	4	5	5	4	4.1
MR125	Roses	Female	ESP	Local	Coastal	Non-native	Joy	60-69	Primary Sch.	14-35	1980s	4	5	4	3	3	1	4	4	4	4	3.6
MR127	Roses	Male	ESP	Local	Coastal	Native	Need	30-39	University	14-35	1990s	4	4	2	2	2	5	5	4	5	5	3.8
WC71	Cadaqués	Male	ESP	Local	Coastal	Native	Strong	40-49	Prof./Tech.	<14	1980s	4	2	1	1	1	5	5	5	5	5	3.4
WC73	Cadaqués	Female	ESP	Local	Coastal	Native	Joy	40-49	University	<14	1980s	4	5	5	5	4	5	3	5	3	5	4.4
ZR37	Roses	Female	FRA	Tourist	Inland	Frequent	Joy	40-49	University	14-35	2000s	2	3	4	2	4	5	3	2	2	5	3.2

				Sociodemographic parameters								Statements (See SM 2 for the entire statement)										
Code	Place of interview	Gender	Country	Local / Tourist	Residence-to-sea distance	Frequency & Antiquity	Marine affinity/ dependence	Age group	Education level	Income	Baseline decade	Hot T°	Marine life	Respect towards nature	Clean coasts	Marine pollution	Boat transit & anchor	Invasive species	Overfish	Nostalgia	Tourism	Average
ZR38	Roses	Male	ESP	Local	Coastal	Non-native	Strong	70-79	University	14-35	1950s	1	4	2	2	4	4	3	4	4	4	3.2
ZR42	Roses	Female	FRA	Tourist	Inland	Frequent	Need	50-59	University	14-35	1980s	1	4	4	2	4	3	4	4	3	3	3.2
MR270	Roses	Male	ESP	Local	Coastal	Native	Strong	18-29	Prof./Tech.	<14	2000s	5	5	3	4	5	5	4	4	4	5	4.4
MR271	Roses	Male	ESP	Local	Coastal	Non-native	Strong	50-59	Prof./Tech.	<14	1970s	3	3	2	3	2	5	5	4	3	5	3.5
AR111	Roses	Male	DEU	Tourist	Inland	Frequent	Joy	50-59	University		2000s	4	3	4	4	3	5	2	2	3	4	3.4
AR113	Roses	Female	CHE	Tourist	Inland	Frequent	Joy	30-39	Prof./Tech.	<14	2000s	4	4	3	2	4	4	4	3	3	4	3.5
AR114	Roses	Female	ESP	Local	Coastal	Native	Strong	18-29	University	<14	2000s	5	5	4	2	5	5	3	5	5	5	4.4
AR115	Roses	Female	ESP	Local	Coastal	Native	Strong	60-69	High Sch.	14-35	1960s	5	5	3	2	5	5	3	4	5	5	4.2
AR116	Roses	Male	ESP	Local	Coastal	Native	Joy	30-39	Prof./Tech.	14-35	1990s	5	5	3	4	5	5	4	4	4	5	4.4
AR120	Roses	Male	FRA	Tourist	Inland	Frequent	Need	50-59	University	35-56	1990s	4	5	2	2	2	4	4	4	4	4	3.5
AR121	Roses	Male	FRA	Tourist	Inland	Frequent	Need	70-79	Middle Sch.	14-35	1980s	4	4	4	2	2	4	4	4	4	4	3.6
AC96	Cadaqués	Male	ESP	Tourist	Near coast	Frequent	Indifferent	40-49	High Sch.	35-56	1980s	5	5	2	5	5	5	3	3	4	5	4.2
AC98	Cadaqués	Female	ESP	Tourist	Coastal	Frequent	Joy	60-69	Master	14-35	1970s	4	4	3	3	5	5	4	4	4	4	4.0
AC99	Cadaqués	Female	BEL	Tourist		Infrequent	Strong	30-39	High Sch.	35-56	1990s	2	1	4	4	5	4	5	5	3	4	3.7
AC100	Cadaqués	Female	ESP	Tourist	Coastal	Infrequent	Joy	50-59	University		2000s	5	4	2	3	3	4	3	3	4	5	3.6
AC101	Cadaqués	Female	ESP	Local	Coastal	Native	Strong	30-39	University	14-35	1990s	5	5	3	2	4	5	3	3	5	5	4.0
AC102	Cadaqués	Female	FRA	Tourist	Inland	Frequent	Need	40-49	Master	14-35	2000s	4	5	2	4	4	5	4	4	5	5	4.2
AC103	Cadaqués	Female	ESP	Tourist	Inland	Frequent	Joy	60-69	University		1990s	5	3	4	2	2	3	3	4	2	5	3.3
WR80	Roses	Female	ESP	Local	Coastal	Native	Joy	50-59	Middle Sch.	14-35	1970s	3	5	2	3	5	4	3	3	4	3	3.5
WR85	Roses	Female	ESP	Local	Coastal	Native	Strong	30-39	Middle Sch.	<14	1990s	4	4	2	3	4	4	3	3	4	2	3.3
WR89	Roses	Male	FRA	Tourist	Inland	Frequent	Need	70-79	Prof./Tech.		1960s	5	4	2	2	3	5	4	3	3	5	3.6
MR79	Roses	Female	ESP	Local	Coastal	Native	Joy	18-29	High Sch.	<14	2000s	4	4	3	3	1	5	5	4	4	5	3.8
MR75	Roses	Male	ESP	Local	Coastal	Native	Strong	60-69		35-56	1960s	3	4	4	2	3	3	4	4	4	5	3.6
MR76	Roses	Female	ESP	Local	Coastal	Native	Strong	18-29	High Sch.	<14	2000s	3	5	2	4	5	5	4	4	4	4	4.0
MR77	Roses	Female	ESP	Local	Coastal	Native	Strong	40-49	Prof./Tech.	14-35	1980s	3	1	5	3	4	4	4	4	4	4	3.6
MR78	Roses	Female	ESP	Local	Coastal	Native	Strong	40-49	University	35-56	1980s	4	5	2	2	4	4	4	4	3	4	3.6
MR79	Roses	Male	ESP	Local	Coastal	Native	Strong	30-39	Prof./Tech.	14-35	1990s	4	4	2	2	3	5	3	5	2	4	3.4
MR80	Roses	Male	ESP	Local	Coastal	Native	Strong	40-49	High Sch.	14-35	1980s	4	5	4	4	4	4	2	4	4	4	3.9
MR81	Roses	Female	ESP	Local	Coastal	Native	Strong	40-49	High Sch.	14-35	1980s	3	5	2	2	5	4	4	3	3	3	3.4
MR83	Roses	Male	ESP	Local	Coastal	Non-native	Strong	60-69	Prof./Tech.	14-35	2000s	5	5	2	5	5	5	3	3	3	4	4.0
MR84	Roses	Female	ESP	Local	Coastal	Native	Need	18-29	Master	14-35	2000s	3	5	3	3	3	4	4	4	4	4	3.7
MR85	Roses	Female	ESP	Local	Coastal	Native	Joy	18-29	High Sch.	No income	2000s	5	4	4	4	3	4	3	4	4	5	4.0
MR86	Roses	Female	ESP	Local	Coastal	Native	Joy	18-29	University	<14	2000s	3	4	3	4	5	4	4	4	5	5	4.1
MR87	Roses	Female	ESP	Local	Coastal	Native	Joy	18-29	High Sch.	<14	2000s	3	4	2	3	5	4	2	4	2	4	3.3
MR88	Roses	Female	ESP	Local	Coastal	Native	Strong	18-29	Prof./Tech.		2000s	3	5	2	3	5	5	4	5	5	4	4.1
MR89	Roses	Female	ESP	Local	Coastal	Native	Joy	40-49	High Sch.	<14	1980s	4	5	4	4	5	1	3	3	5	5	3.9
MR90	Roses	Female	ESP	Local	Coastal	Native	Joy	40-49	University	14-35	1980s	4	3	4	3	4	4	3	3	3	5	3.6
AR56	Roses	Female	ESP	Local	Coastal	Native	Strong	40-49	Prof./Tech.		1980s	3	5	3	3	5	3	3	3	3	3	3.4
AR58	Roses	Male	ESP	Local	Coastal	Native	Strong	18-29	Prof./Tech.	<14	2000s	4	3	3	5	5	4	3	4	3	4	3.8
AR60	Roses	Male	ESP	Local	Coastal	Native	Strong	18-29	Prof./Tech.	<14	2000s	5	5	5	5	5	5	2	5	5	4	4.6
AR61	Roses	Male	ESP	Local	Coastal	Native	Joy	18-29	Prof./Tech.	<14	2000s	4	4	3	5	5	4	4	5	4	5	4.3
AR64	Roses	Female	ESP	Tourist	Inland	Infrequent	Joy	18-29	University	No income	1990s	4	4	4	4	4	4	4	4	4	5	4.1
AR68	Roses	Female	ESP	Local	Coastal	Native	Joy	40-49	High Sch.	<14	1980s	5	5	4	3	4	4	3	3	4	3	3.8
AR69	Roses	Male	ESP	Local	Coastal	Non-native	Strong	50-59	High Sch.	14-35	1970s	1	5	3	2	4	5	4	3	3	4	3.4
AR70	Roses	Female	ESP	Local	Coastal	Native	Strong	50-59	Middle Sch.	14-35	1970s	4	5	4	4	5	4	4	4	5	5	4.4
AR71	Roses	Female	ESP	Local	Coastal	Non-native	Joy	70-79	No Sch.	No income	1960s	2	4	3	2	2	4	3	4	4	2	3.0
AR72	Roses	Female	ESP	Tourist	Coastal	Frequent	Strong	50-59	Prof./Tech.	35-56	1980s	5	4	2	3	2	2	4	4	5	5	3.6
MP40	Port Selva	Female	ESP	Local	Coastal	Native	Joy	18-29	Middle Sch.	No income	2000s	4	4	3	4	4	3	3	3	4	2	3.4
MP41	Port Selva	Female	ESP	Local	Near coast	Native	Strong	50-59	PhD	14-35	1970s	4	5	5	3	5	5	4	4	4	5	4.4
MP42	Port Selva	Female	ESP	Local	Coastal	Native	Joy	40-49	Prof./Tech.	14-35	1980s	3	4	4	5	2	4	4	5	3	4	3.8
MP44	Port Selva	Female	ESP	Local	Coastal	Native	Joy	40-49	Prof./Tech.	<14	1980s	4	5	3	4	5	5	1	1	5	5	3.8
MP45	Port Selva	Female	ESP	Local	Coastal	Native	Strong	50-59	University	14-35	1970s	4	5	4	3	1	1	3	4	5	5	3.5
MP46	Port Selva	Female	ESP	Local	Coastal	Native	Need	>90	University	14-35	1930s	3	5	4	2	5	2	4	4	5	4	3.8
MP47	Port Selva	Female	ESP	Local	Coastal	Native	Joy	18-29	Master	<14	2000s	3	4	2	3	4	5	3	3	4	4	3.5
MP49	Port Selva	Female	ESP	Tourist	Near coast	Frequent	Indifferent	18-29	Middle Sch.	No income	2000s	4	5	4	3	5	3	4	4	1	5	3.8
MP50	Port Selva	Male	ESP	Local	Coastal	Native	Strong	18-29	Prof./Tech.	<14	2000s	2	5	4	4	5	3	4	1	4	3	3.5
MP51	Port Selva	Female	ESP	Local	Coastal	Native	Strong	40-49	Middle Sch.	14-35	1980s	5	3	2	2	1	4	4	3	5	2	3.1
MP53	Port Selva	Female	ESP	Tourist	Inland	Frequent	Strong	40-49	Master	>56	1990s	5	4	3	5	5	4	4	3	4	4	4.1
MC50b	Cadaqués	Female	ESP	Local	Coastal	Native	Strong	30-39	High Sch.	14-35	1990s	3	5	4	2	3	5	4	4	4	5	3.9
MC51b	Cadaqués	Female	ESP	Local	Coastal	Native	Strong	18-29	Middle Sch.	<14	2000s	2	4	5	5	5	5	3	2	3	4	3.8
MC52b	Cadaqués	Male	ESP	Local	Coastal	Native	Strong	40-49	University	14-35	1980s	4	4	2	4	4	5	3	4	5	4	3.9
MC54	Cadaqués	Female	ESP	Local	Coastal	Native	Strong	50-59	Middle Sch.	14-35	1970s	4	5	3	4	5	5	5	5	5	5	4.6
MC55	Cadaqués	Female	ESP	Local	Coastal	Native	Indifferent	60-69	High Sch.		1960s	3	5	5	5	5	5	5	5	3	5	4.6
MC56	Cadaqués	Female	ESP	Local	Coastal	Non-native	Strong	60-69	University	14-35	1980s	5	5	2	4	5	5	4	5	4	5	4.4
MC58	Cadaqués	Male	ESP	Local	Coastal	Native	Strong	40-49	University	35-56	1980s	3	5	4	3	3	3	5	5	5	5	4.1
MC59	Cadaqués	Male	ESP	Tourist	Coastal	Frequent	Need	30-39	Master	<14	2000s	3	3	4	3	3	4	4	3	3	5	3.5
MC60	Cadaqués	Male	ESP	Tourist	Inland	Frequent	Need	30-39	Master	<14	2000s	4	5	3	3	3	5		3	3	5	3.8
MC61	Cadaqués	Male	ESP	Local	Coastal	Native	Indifferent	18-29	Middle Sch.	No income	2000s	3	2	3	4	4	3	4	3	2	5	3.3
MC62	Cadaqués	Female	ESP	Local	Coastal	Native	Joy	18-29	High Sch.	No income	2000s	4	4	3	4	2	5	3	3	4	5	3.7
WP01	Port Selva	Male	ESP	Local	Coastal	Native	Joy	70-79	Master	35-56	1950s	3	5	2	2	2	5	3	3	5	4	3.4
WP02	Port Selva	Male	FRA	Tourist	Inland		Joy	60-69	Prof./Tech.	<14		3	5	1	2	4	5	2	5	4	5	3.6
WP05	Port Selva	Male	GBR	Tourist	Near coast	Frequent	Joy	30-39	Prof./Tech.	14-35	1980s	4	4	3	2	5	3	3	2	3	4	3.3
WP06	Port Selva	Male	ESP	Tourist	Coastal	Frequent	Joy	70-79	Master	35-56	1950s	5	5	5	3	5	4	4	5	5	4	4.5
WR38	Roses	Male	ESP	Local	Coastal	Non-native	Joy	18-29	Middle Sch.	<14	2000s	2	5	1	4	5	4	3	4	5	3	3.6
WR40	Roses	Male	ESP	Local	Coastal	Non-native	Strong	50-59	High Sch.	<14	1990s	3	3	5	2	3	4	4	3	4	4	3.5
WR42	Roses	Male	ESP	Local	Coastal	Non-native	Indifferent	30-39	Middle Sch.		1990s	3	3	5	3	5	4	4	3	5	5	4.0
WR43	Roses	Female	ESP	Local	Coastal	Non-native	Joy	40-49	Prof./Tech.		1980s	2	5	4	2	5	3	3	4	5	3	3.6
WR45	Roses	Male	FRA	Tourist	Inland	Frequent	Joy	50-59	Prof./Tech.	14-35	1980s	1	5	2	2	3	4	4	4	4	4	3.3
WR46	Roses	Male	ESP	Local	Coastal	Native	Strong	18-29	High Sch.	14-35	2000s	4	5	3	4	5	5	3	4	3	4	4.0
WR47	Roses	Female	ESP	Local	Coastal	Native	Need	50-59	Middle Sch.		1970s	5	5	3	1	5	1	1	1	5	5	3.2
WR48	Roses	Female	ESP	Tourist	Coastal	Frequent	Need	18-29	Master	14-35	1990s	5	3	4	4	4	2	4	3	5	5	3.9
WR49	Roses	Female	ESP	Local	Coastal	Native	Joy	18-29	University	<14	2000s	3	5	5	4	4	4	3	5	3	4	4.0
WR50	Roses	Male	ESP	Tourist	Near coast	Frequent	Joy	40-49	Master	14-35	1980s	4	5	4	5	5	5	3	3	3	4	4.1
WR52	Roses	Male	ESP	Local	Coastal	Non-native	Strong	60-69	High Sch.		2000s	1	5	1	1	4	5	5	5	1	1	2.9
AC32	Cadaqués	Female	BEL	Tourist	Near coast	Frequent	Need	18-29	Prof./Tech.	14-35	1990s	3	5	2	2	4	4	4	1	1	4	3.0
AC35	Cadaqués	Female	ESP	Local	Coastal	Native	Joy	60-69	University	35-56	1960s	5	5	5	4	5	1	3	2	5	3	3.8
AC36	Cadaqués	Female	FRA	Tourist	Inland	Frequent	Indifferent	70-79	University	14-35	2000s	5	3	1	4	3	4	3	3	3	4	3.3
AC37	Cadaqués	Male	ESP	Tourist	Coastal	Frequent	Need	70-79	University	14-35	1960s	5	5	2	2	4	5	5	5	4	5	4.2
AC40	Cadaqués	Female	ESP	Local	Coastal	Native	Joy	50-59	University	14-35	1970s	5	5	2	3	5	5	5	5	5	5	4.5
AC41	Cadaqués	Female	ESP	Local	Coastal	Native	Joy	40-49	Primary Sch.		1980s	2	5	2	3	5	5	1	2	5	2	3.2
AC42	Cadaqués	Female	ESP	Local	Coastal	Native	Strong	18-29	University	<14	2000s	5	4	3	4	5	5	3	4	4	4	4.1
AC44	Cadaqués	Female	ESP	Local	Coastal	Native	Strong	30-39	University	14-35	1990s	3	5	5	5	5	5	3	4	4	5	4.4
AC45	Cadaqués	Male	ESP	Tourist	Near coast	Frequent	Need	30-39	Master	35-56	1980s	4	4	2	3	5	4	3	4	3	4	3.6
AC49	Cadaqués	Female	ESP	Tourist	Near coast	Frequent	Joy	50-59	University	14-35	1970s	4	4	3	2	3	4	3	3	3	5	3.4
AC51	Cadaqués	Male	ESP	Local	Coastal	Native	Indifferent	40-49	High Sch.	14-35	1980s	4	3	4	2	1	1	1	2	5	1	2.4
AC53	Cadaqués	Female	ESP	Local	Coastal	Native	Joy	40-49	University	14-35	1980s	4	4	2	2	4	2	3	2	5	3	3.1
AC55	Cadaqués	Female	ESP	Local	Coastal	Native	Need	60-69	University		1960s	1	3	4	2	4	2	4	2	2	4	2.8
WC20	Cadaqués	Female	FRA	Tourist	Inland	Frequent	Joy	60-69	Middle Sch.	14-35	2000s	2	3	3	2	4	5	3	4	3	4	3.3
WC21	Cadaqués	Female	FRA	Tourist	Inland	Infrequent	Strong	50-59	High Sch.	14-35	2000s	4	5	3	5	5	4	5	4	5	5	4.5
WC22	Cadaqués	Male	FRA	Tourist	Inland	Infrequent	Strong	50-59	Prof./Tech.	14-35	2000s	3	5	2	2	5	4	4	5	4	4	3.8
WC25	Cadaqués	Female	FRA	Tourist	Inland	Infrequent	Joy	60-69	Prof./Tech.	14-35	2000s	1	5	2	2	5	5	4	3	2	3	3.2
WC27	Cadaqués	Male	NLD	Tourist	Near coast	Frequent	Joy	60-69	Prof./Tech.	>56	2000s	4	4	2	2	4	2	3	4	3	4	3.2
WC29	Cadaqués	Female	ESP	Tourist	Near coast	Frequent	Joy	40-49	University		1990s	5	5	4	4	4	5	5	5	4	5	4.6
WC30	Cadaqués	Female	ESP	Tourist	Near coast	Infrequent	Joy	50-59	Master	14-35	1990s	3	4	2	2	5	4	4	3	5	4	3.6
WC33	Cadaqués	Female	ESP	Local	Coastal	Native	Strong	30-39	University		1990s	5	5	5	4	5	5	4	4	5	5	4.7
WC34	Cadaqués	Female	ESP	Local	Coastal	Native	Joy	80-89	Primary Sch.		1940s	3	5	3	4	5	5	5	5	5	5	4.5
WC35	Cadaqués	Male	ESP	Local	Coastal	Native	Indifferent	70-79	Primary Sch.	35-56	1950s	1	5	2	2	5	5	5	5	1	3	3.4
WC37	Cadaqués	Male	ESP	Local	Coastal		Joy	60-69	High Sch.	<14		4	4	1	2	2	3	3	4	3	4	3.0

Coastal marine habitats deterioration. Perception of Cap de Creus Marine Protected Area (NE Spain) users. Regional Environmental Change. Miguel Mallo, Patrizia Ziveri, Sergio Rossi, Victoria Reyes-García. Corresponding authors: Miguel Mallo (miguelmallo91@gmail.com), Patrizia Ziveri (Patrizia.ziveri@uab.cat). Institut de Ciència i Tecnologia (ICTA). Universitat Autònoma de Barcelona (UAB), Bellaterra, Barcelona, Spain.
