# Supplementary figures and images for: Coastal marine habitats deterioration according to users’ perception: the case of Cap de Creus Marine Protected Area (NE Spain)
Source: Reg Environ Change. 2024 Oct 10;24(4):155. doi: 10.1007/s10113-024-02322-4 (PMC11467071; doi:10.1007/s10113-024-02322-4)

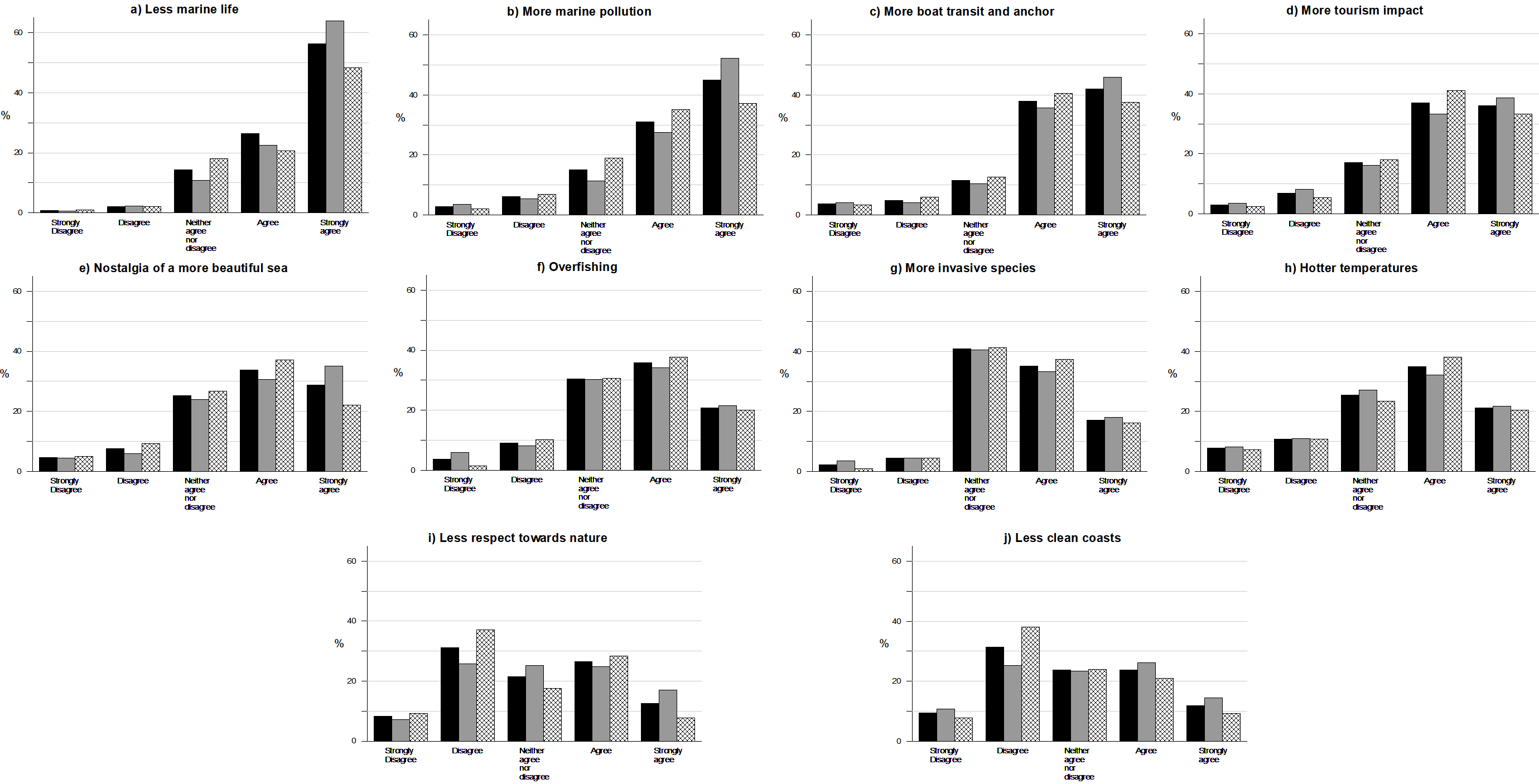

Supplement: Supplementary file 5 — Supplementary file5 Online Resource 5. Distribution of responses to 10 Likert scale statements. (PNG 55 KB) [file 10113_2024_2322_MOESM5_ESM.png]
