# Supplementary material for: Coastal marine habitats deterioration according to users’ perception: the case of Cap de Creus Marine Protected Area (NE Spain)
Source: Reg Environ Change. 2024 Oct 10;24(4):155. doi: 10.1007/s10113-024-02322-4 (PMC11467071; doi:10.1007/s10113-024-02322-4)
Supplement: Supplementary file 6 — Supplementary file6 Online Resource 6. Spider diagrams comparing average values of responses to Likert scale statements, by subsamples differentiating between locals and tourists with different characteristics. Concentric polygons represent the axis of the average values, where 1 = “strongly disagree”, 2 = “disagree”, 3 = “neither agree nor disagree”, 4 = “agree”, and 5 = “strongly agree”. Each spike represents an abbreviation of each statement in the Likert table (see Online Resource 2). Statements highlighted in yellow have averages with statistically significant differences between local and tourist subsamples in a Kruskal-Wallis rank sum test (Table 2). In italics the subsamples for which we found statistically significant differences in a post-hoc Dunn test (p-value inside parenthesis; (*) p < 0.05, (**) p < 0.01). (PDF 517 KB) [file 10113_2024_2322_MOESM6_ESM.pdf]

Online Resource 5

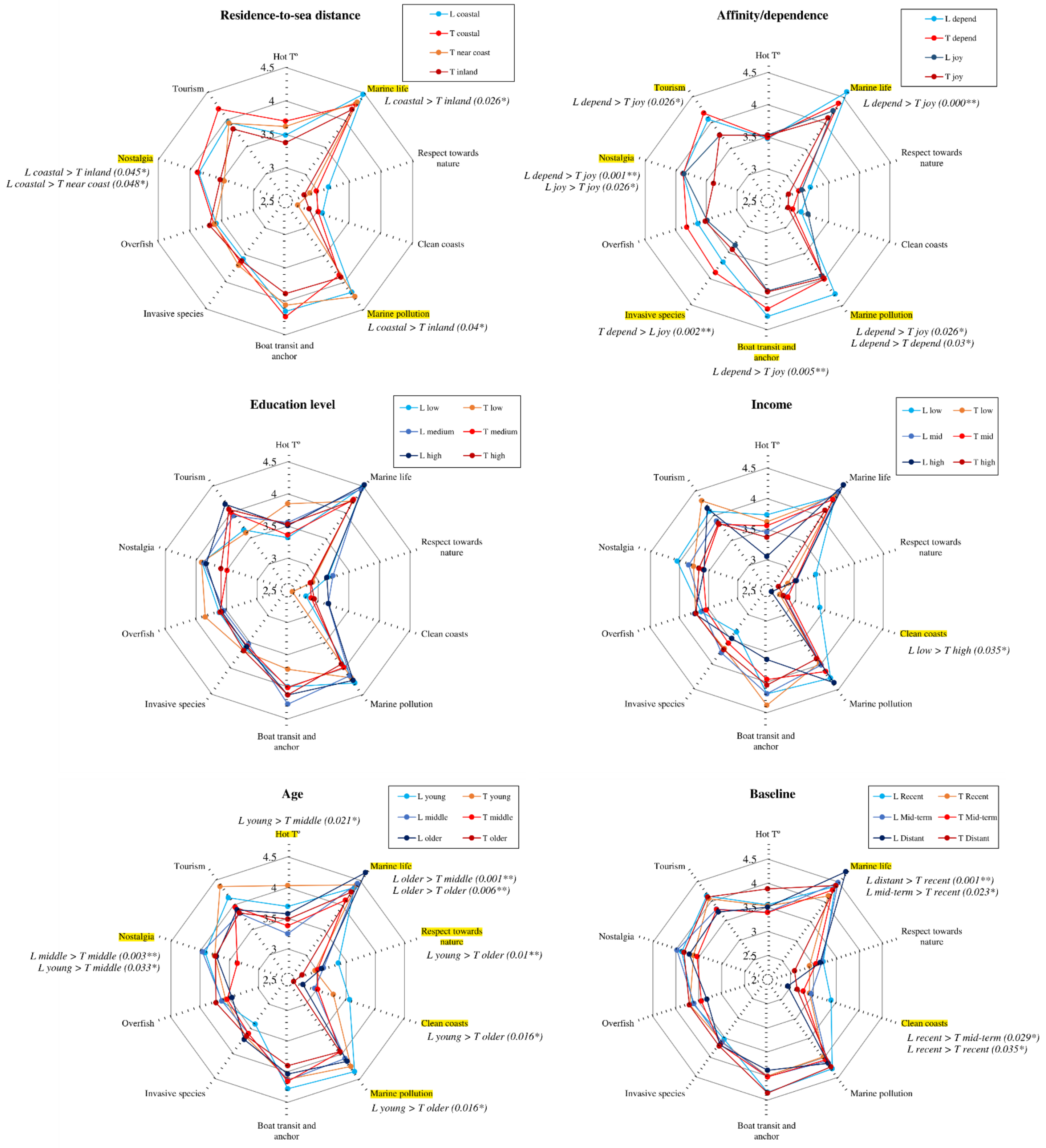

Coastal marine habitats deterioration. Perception of Cap de Creus Marine Protected Area (NE Spain) users. Regional Environmental Change. Miguel Mallo, Patrizia Ziveri, Sergio Rossi, Victoria Reyes-García. Corresponding authors: Miguel Mallo (miguelmallo91@gmail.com), Patrizia Ziveri (Patrizia.ziveri@uab.cat). Institut de Ciència i Tecnologia (ICTA). Universitat Autònoma de Barcelona (UAB), Bellaterra, Barcelona, Spain.
